# Glycolytic suppression of cell adhesion promotes thyroid morphogenesis in response to nutrition

**DOI:** 10.1038/s44319-026-00868-4

**Published:** 2026-07-20

**Authors:** Asako Shindo, Shiho Nishiguchi, Ayaka Fujiwara, Kaoru Nakashima

**Affiliations:** 1 Department of Biological Sciences, Graduate School of Science, The University of Osaka, Toyonaka, Japan; 2 Institute of Molecular Embryology and Genetics, Kumamoto University, Kumamoto, Japan

**Keywords:** Development, Metabolism

## Abstract

Organ morphogenesis relies on collective cellular behaviors. While intrinsic developmental programs governing cellular behaviors are well characterized, the effects of environmental and systemic factors on these behaviors remain poorly understood. Here, we investigate how nutritional availability modulates cellular behaviors during thyroid morphogenesis using *Xenopus* tadpoles as an in vivo model. RNA-seq analysis of thyroid regions from fed and unfed tadpoles shows that nutritional status alters gene expression profiles. We identify cell adhesion-related genes as key targets, with expression increased in unfed tadpoles and decreased in fed tadpoles. Immunostaining for E-cadherin supports the RNA-seq results, and functional inhibition of E-cadherin facilitates thyroid follicle formation. We further demonstrate that glycolytic activity reduces junctional E-cadherin accumulation in the thyroid, linking metabolism to the regulation of cell adhesion. Lastly, thyroid cells forming multi-luminal structures exhibit distinct apical-basal polarity, and these structures are enhanced by glycolytic activity and suppression of cell adhesion. These findings reveal a mechanism by which nutritional status remodels cellular properties underlying collective cellular behaviors during organ morphogenesis.

## Synopsis

Nutritional status regulates thyroid morphogenesis in vivo through glycolysis-associated suppression of cell adhesion, providing a link between metabolic state and epithelial tissue architecture during organogenesis.

- Cell adhesion-related genes are upregulated in unfed *Xenopus* tadpoles during thyroid morphogenesis.
- Thyroid follicle cells frequently establish multiple apical domains during follicle formation.
- Glycolysis-associated reduction of cell adhesion promotes thyroid morphogenesis.

## Abstract (web summary)

Nutritional status regulates thyroid morphogenesis in vivo through glycolysis-associated suppression of cell adhesion, providing a link between metabolic state and epithelial tissue architecture during organogenesis.

## Graphical abstract

**Figure:**
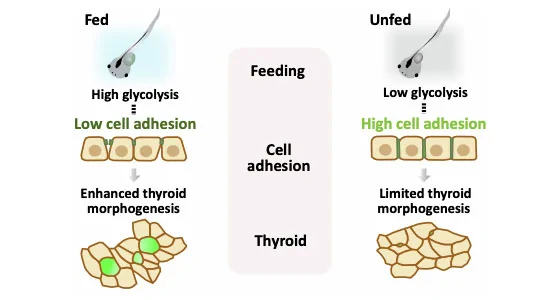

**Figure:**
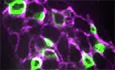

## Introduction

Morphogenesis has long been viewed as a tightly regulated, gene-driven process governed predominantly by endogenous programs. This framework has significantly contributed to advances in developmental biology (Harland and Grainger, 2011; Charney et al, 2017). Building on this foundation, increasing evidence suggests that external cues can influence morphogenesis, indicating that the process is responsive to environmental influences and may not be entirely autonomous (Neitzel et al, 2025; Eroshkin et al, 2024; Ripley et al, 2023; Romney et al, 2018). Among these exogenous cues, the nutritional environment is one of the most potent factors capable of modulating gene expression and cellular behavior during morphogenesis. For instance, studies using vertebrate models have identified specific nutrients and metabolic pathways crucial for morphogenesis (Takata et al, 2023; Cao et al, 2024; Oginuma et al, 2017). Environmental nutrients have also been shown to affect not only gene expression but also the timing of organogenesis (Takagishi et al, 2022; McKeown and Cline, 2019; McKeown et al, 2017). These findings collectively point to non-autonomous regulation as a fundamental mechanism in morphogenesis. Understanding how environmental signals such as nutrients are integrated with genetic programs is therefore crucial for understanding organ morphogenesis.

One potential mechanism of such non-autonomous regulation is the modulation of collective cell behaviors through changes in cell adhesion, cytoskeletal dynamics, and the extracellular matrix (ECM) during morphogenesis. During morphogenesis, cells undergo shape changes and move collectively, processes that require dynamic regulation of intracellular molecules, including cell adhesion molecules and the cytoskeleton (Weng et al, 2022; Shindo and Wallingford, 2014). Several metabolic pathways have been shown to regulate these cellular components in the context of cell biology. For example, glucose uptake and glycolysis reorganize the actin cytoskeleton, thereby facilitating insulin secretion in pancreatic cells and stimulating immune cell migration (Kishore et al, 2017; Liu et al, 2022). Additionally, repression of mTORC1 under nutrient deprivation has been reported to promote ECM degradation in breast cancer cells (Colombero et al, 2021). These findings suggest that external nutrients may regulate collective cell behaviors by modulating downstream cellular machinery during morphogenesis and beyond. However, whether environmental nutrients regulate the collective cell behaviors that physically shape organs and tissues during vertebrate morphogenesis remains largely unknown.

*Xenopus laevis* is a well-established amphibian model that offers advantages for investigating the role of nutrients during organ morphogenesis. In this animal, feeding begins during a period of active organogenesis, and we previously reported that thyroid morphogenesis progresses in a feeding-dependent manner (Takagishi et al, 2022). An important advantage of using *Xenopus* tadpoles is that nutritional conditions during organogenesis can be experimentally manipulated through straightforward methods such as altering food composition or restricting nutrient intake. Furthermore, the optical transparency of tadpoles allows high-resolution imaging of internal organs (Voigt et al, 2024; Takagishi et al, 2022). This combination of nutritional manipulation and imaging provides a powerful platform for uncovering molecular and cellular mechanisms that govern developmental processes, including regeneration (Williams et al, 2021).

The thyroid gland is composed of multiple follicles, a structure indispensable for its endocrine function. Thyroid hormones are synthesized and stored within the thyroglobulin-rich colloid of follicular lumen and are released into the bloodstream after reuptake and processing by follicular epithelial cells. Each follicular lumen is surrounded by a single layer of epithelial cells, which usually exhibit a well-defined apical-basal polarity, as observed in studies of spherical and tubular epithelial tissues (Horikoshi et al, 2009; Thottacherry et al, 2023). Importantly, thyroid hormones are essential for the development of multiple organs, including the lung (Archavachotikul et al, 2002), intestines (Tanizaki et al, 2022), neurons and brain (De Escobar et al, 2004; Patel et al, 2011), as well as for organ regeneration (Hirose et al, 2019; Francisco, 2022). Thus, the proper thyroid morphogenesis is critical for overall organ development. Despite its significance, the cellular and molecular mechanisms underlying thyroid morphogenesis remain unclear.

A key feature of thyroid follicles is the organization of follicular epithelial cells around a lumen, a process that requires the establishment of apical-basal cell polarity. In structures with a single lumen, such as MDCK cysts or the intestinal tube, epithelial cells establish a single apical-basal polarity axis and orient their apical surfaces toward the lumen. Knockdown of apical polarity markers such as *par3*, or depletion of *β1-integrin*, induces multi-axial polarity by disturbing apical domain development or cell-ECM interactions, respectively (Akhtar and Streuli, 2013; Horikoshi et al, 2009). These findings suggest that disruption of apical-basal polarity can lead to the emergence of cells with multiple polarity axes during luminal tissue formation. Interestingly, multi-axial cells and multi-luminal structures can also arise spontaneously without any genetic perturbation, both in vivo and in vitro (Bebelman et al, 2024; Lu et al, 2025). Notably, *Xenopus* thyroid epithelial cells exhibit multiple lumina during the early phase of follicle morphogenesis following the onset of feeding (Takagishi et al, 2022), suggesting a potential link between nutrient availability and the regulation of apical-basal cell polarity.

In this study, we used *Xenopus laevis* tadpoles to investigate how external nutrients regulate cellular properties during thyroid morphogenesis. We performed RNA-seq analyses to compare gene expression in the thyroid region between fed and unfed tadpoles. In addition to substantial changes in metabolism-related genes, cell adhesion molecules were upregulated in the thyroid region of unfed tadpoles and downregulated in fed tadpoles. Localization analysis and functional inhibition of the cell adhesion molecule E-cadherin (Cdh1) indicated that reduced cell adhesion facilitates thyroid morphogenesis. We further identified glycolysis as a potential regulator of E-cadherin, demonstrating that glucose-enriched diets reduce E-cadherin accumulation at cell–cell contacts in the thyroid. Consistently, pharmacological inhibition of glycolysis following feeding increased E-cadherin accumulation. These findings highlight a role for intracellular responses to specific nutrients in regulating organ morphogenesis.

## Results

### Feeding alters gene expression in the thyroid tissue region

**Figure 1:**
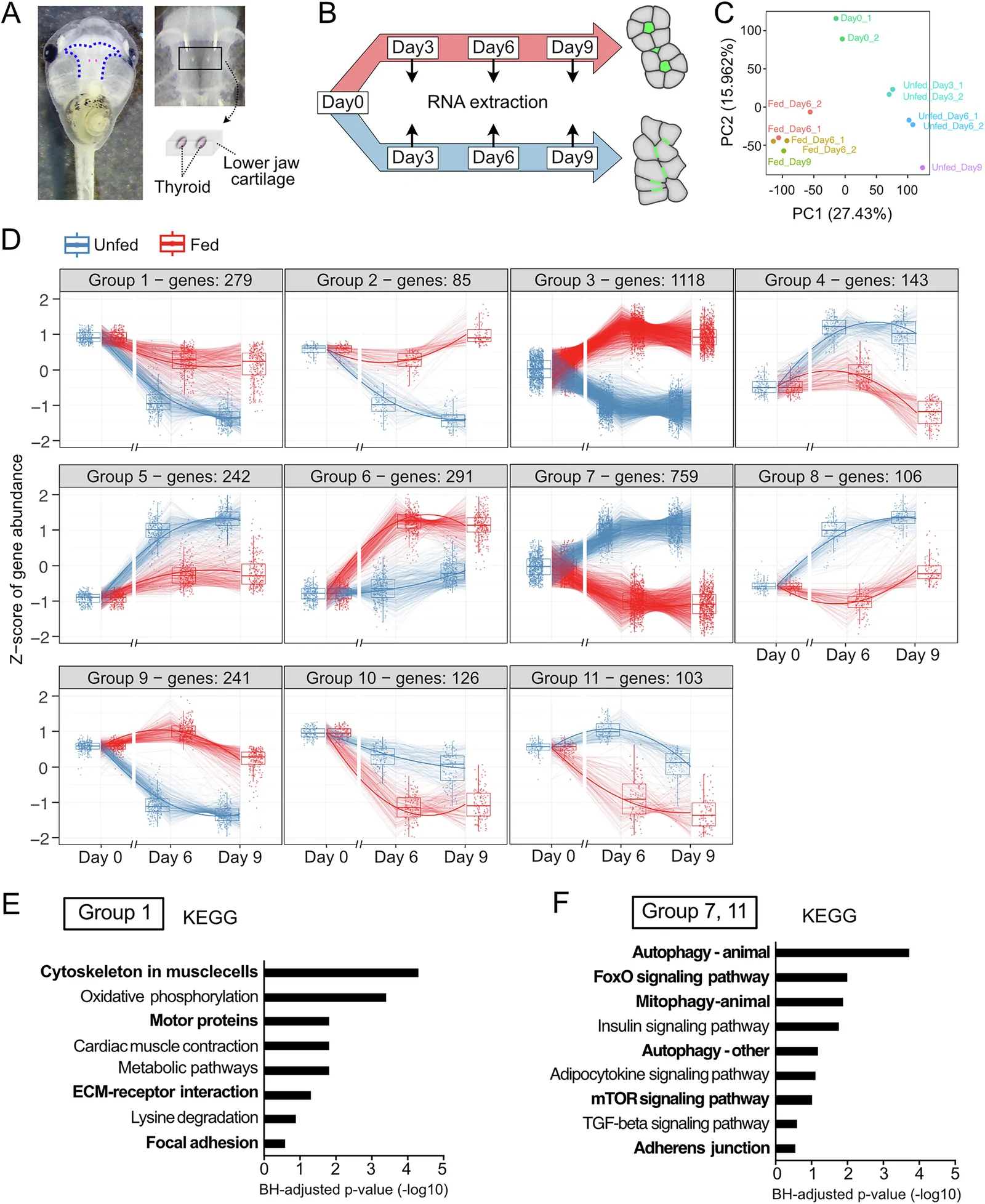
Feeding alters gene expression in the thyroid region. (**A**) Ventral view of a fixed *Xenopus laevis* tadpole showing the location of the thyroid glands. Blue dotted lines outline the lower jaw cartilage, and solid pink ovals mark the left and right thyroid glands. The black rectangle indicates the region isolated for RNA-seq analysis. Both the left and right thyroid glands are included in the isolated tissue piece, along with surrounding lower jaw cartilage. (**B**) Timing of RNA isolation from fed tadpoles after the onset of feeding and from unfed tadpoles at the matched time points. Day 0 is defined as stage 46. (**C**) Principal component analysis (PCA) plot of RNA-seq data from isolated tissue piece containing the thyroid. (**D**) Clustering analysis based on gene expression dynamics using Day 0, Day 6, and Day 9 samples. Day 3 samples were excluded from the analysis because biological replicates showed relatively inconsistent expression patterns. Red lines indicate gene sets from fed tadpoles, and blue lines indicate those from unfed tadpoles. The vertical axis represents expression levels, and the horizontal axis represents the time points of RNA isolation. Center lines indicate the medians; box limits indicate the 25th and 75th percentiles; whiskers indicate the most extreme values within 1.5 × the interquartile range; points beyond the whiskers are plotted as outliers. All numerical values are provided in Source Data file. (**E**, **F**) KEGG pathway analysis of genes categorized in Group 1, 7, and 11. The horizontal axis represents enrichment significance, shown as −log10(BH-adjusted *P* values). Source data are available online for this figure.

**Figure EV1:**
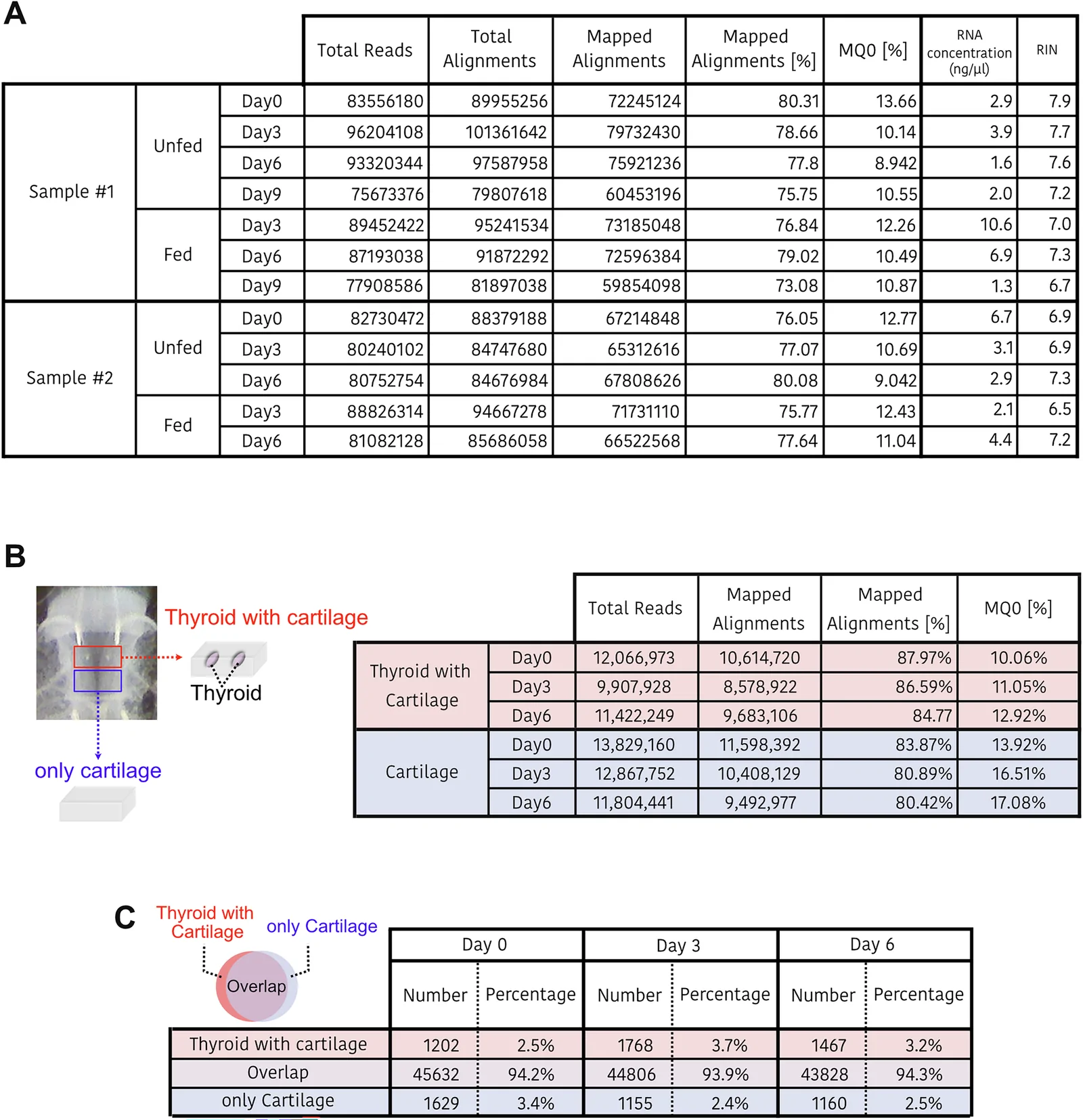
Related to Fig. 1. (**A**) Summary of RNA-seq quality metrics generated in this study. The listed parameters include total reads, total alignments, mapped alignments, the percentage of mapped alignments, the percentage of reads with mapping quality 0 (MQ0), RNA concentration, and RNA integrity number (RIN). (**B**) The thyroid or cartilage region used for the RNA-seq analysis. The table shows RNA-seq quality metrics used for comparison between two different regions, thyroid with cartilage and cartilage. (**C**) The table displaying the overlap of expressed genes in the thyroid region (red) and cartilage region (blue) at Days 0, 3, and 6. RNA was extracted from unfed larvae, and genes with raw counts ≤5 were excluded from the analysis. Percentages represent the proportion of genes in each category relative to the total number detected at each time point.

To perform RNA-seq analysis, the thyroid gland together with the surrounding lower jaw cartilage was dissected as a single block of tissue from tadpoles at Day 0, corresponding to stage (st.) 46, when tadpoles begin swimming and feeding (Fig. 1A). RNA was then extracted from the isolated tissues, and additional samples were collected at Day 3, Day 6, and Day 9 from both fed and unfed groups (Figs. 1B and EV1A). Since the thyroid gland at these stages is embedded within the lower jaw cartilage, we chose to perform RNA-seq on the dissected tissue block containing both the thyroid and surrounding cartilage, as an initial approach to examine gene expression in this region. To confirm whether RNA-seq analysis can detect thyroid-specific genes from the heterogeneous tissue blocks, we also performed RNA-seq on cartilage tissue isolated from the region posterior to the thyroid in the unfed group, and compared the results with those from the thyroid-containing tissue (Fig. EV1B). Over 90% of expressed genes were shared between the two tissue blocks (Fig. EV1C). A small proportion of the genes (2.5–3.4%) were specifically expressed in either the thyroid region or the lower jaw cartilage, and the thyroid-specific gene *thyroglobulin* was detected in the thyroid-containing tissue (GO terms are in Fig. EV4). These results suggest that our RNA-seq analysis can reliably detect thyroid-specific gene expression despite the heterogeneous tissue composition.

**Figure EV2:**
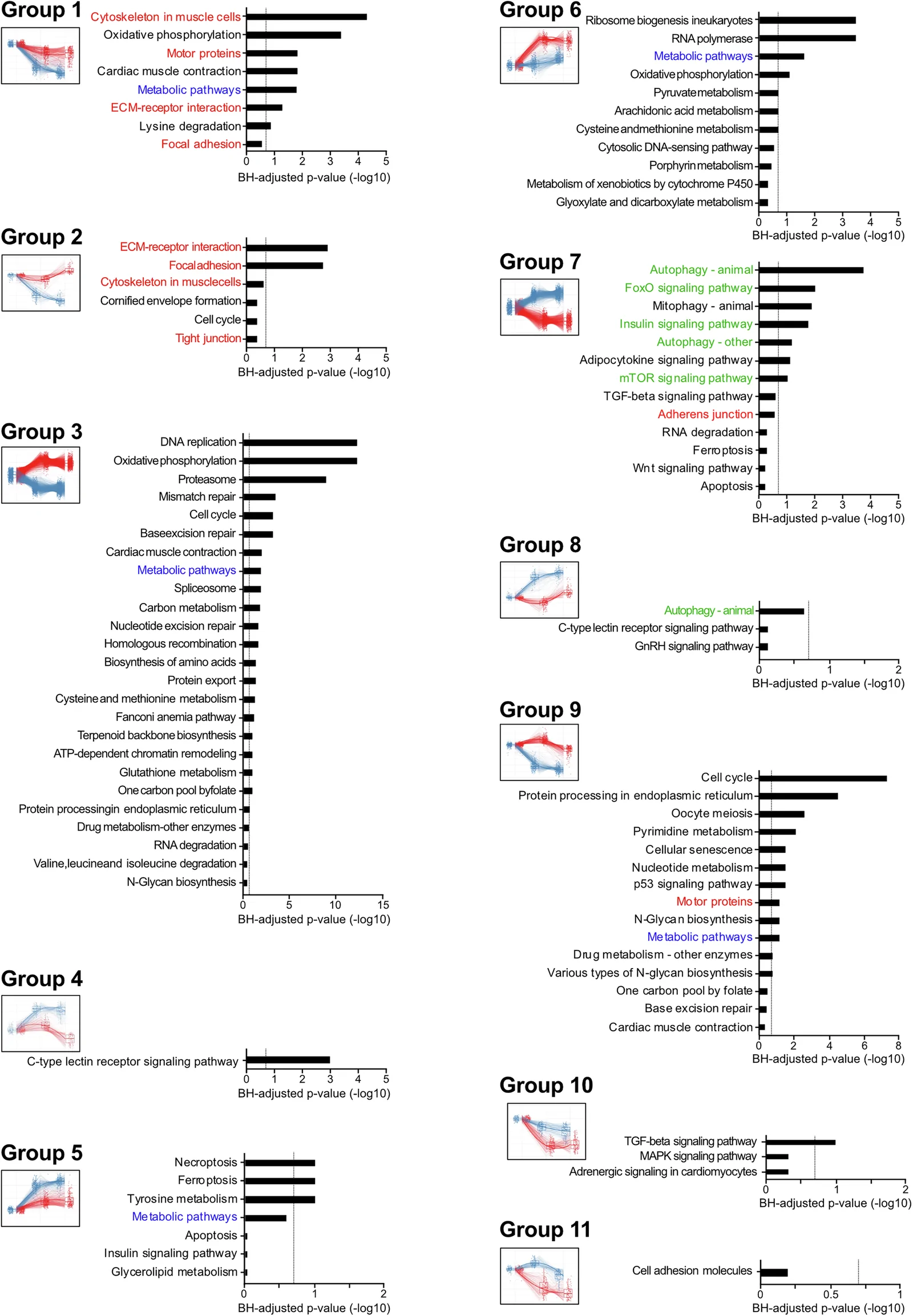
Related to Figs. 1 and 2. KEGG pathway analysis for each group in Fig. 1D. Bar plots show enriched pathways (*x* axis: −log10(BH-adjusted *P* values) for representative groups. Selected pathways are color-coded to highlight categories related to cell behaviors (red), metabolism (blue), and starvation-related genes (green).

Principal component analysis (PCA) of RNA-seq data, performed with duplicate samples at Day 0, 3, and 6 and a single sample at Day 9, demonstrates that gene expression profiles differ between the fed and unfed groups (Fig. 1C). To categorize genes based on temporal changes in expression from Day 0 to Day 9, we performed clustering analysis using RNAseqChef (Etoh and Nakao, 2023) on genes that showed differential expression between fed and unfed conditions at Day 6 (fold change ≥1.5, FDR ≤ 0.1). As a result, 13 groups were identified based on temporal expression patterns (Dataset EV1), and of which 11 representative groups are shown in Fig. 1D. KEGG pathway analysis shows that the genes associated with metabolic pathways were enriched in Group 1, 3, 6, and 9, which has genes upregulated in the fed and downregulated in the unfed (Figs. 1D and EV2). On the other hand, the genes involved in autophagy and FoxO signaling, typically activated by starvation, were grouped in Group 7 (Fig. EV2). Group 5 also includes genes involved in metabolic pathways, such as fructose-bisphosphatase and glycogen phosphorylase, which are associated with metabolic adaptation to fasting. Furthermore, pathway analysis revealed that genes associated with the cytoskeleton and ECM, which are significant for collective cell behaviors and morphogenesis, were upregulated in the fed condition, as shown in Groups 1 and 2 (Figs. 1E and EV2). Also, genes related to cell adhesion were in Group 4, 7, and 11, which were upregulated in the unfed and downregulated in the fed (Figs. 1F,  EV1, and EV2).

### Feeding and starvation differentially influence the expression of genes related to cell adhesion, extracellular matrix, and cytoskeleton

**Figure 2:**
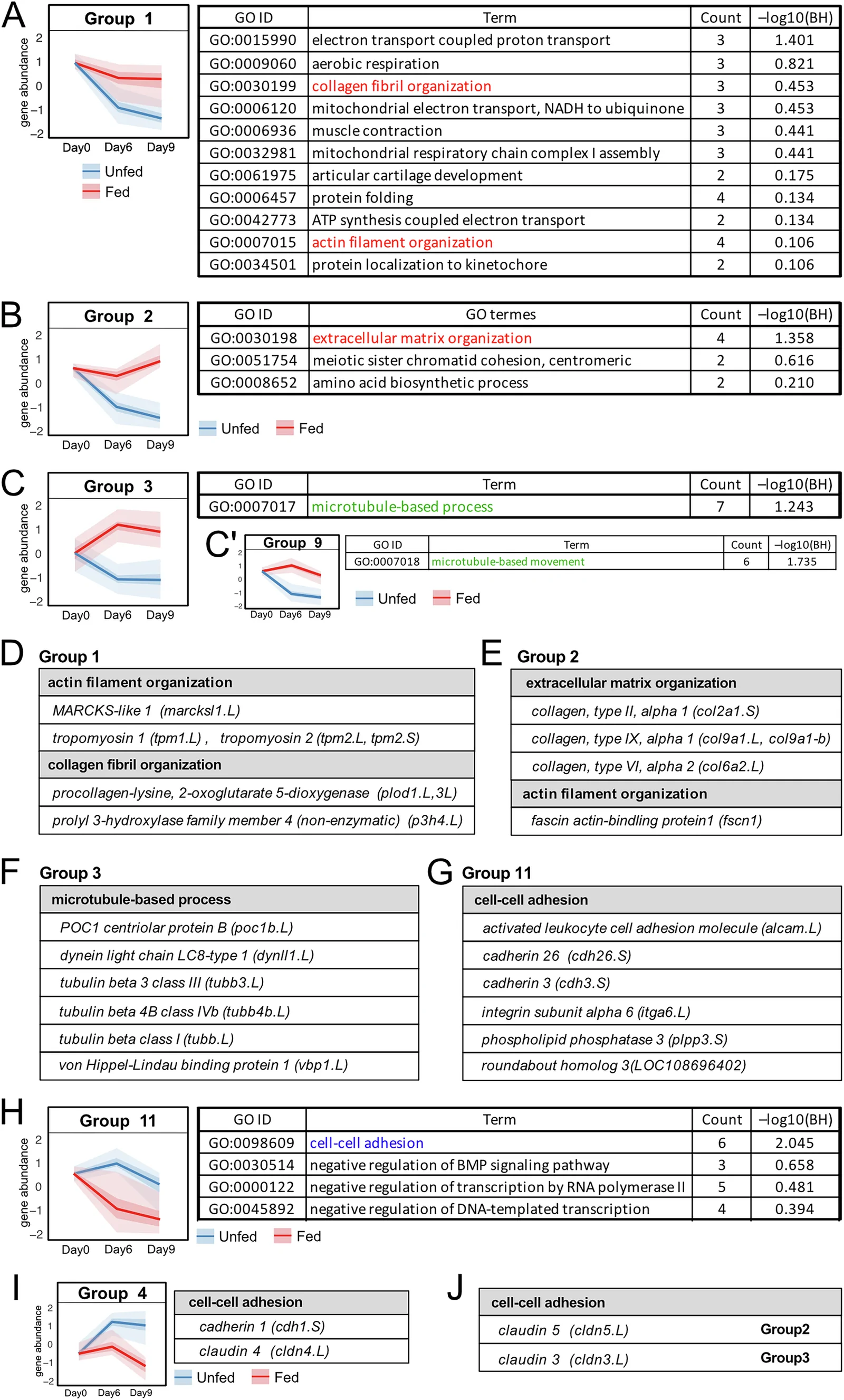
Feeding and starvation diff erentially infl uence the expression of genes related to cell adhesion, extracellular matrix,and cytoskeleton. (**A**) Gene ontology (GO) analysis of genes categorized in Group 1. Actin and ECM-associated terms are labeled in red. (**B**) GO analysis of genes categorized in Group 2. ECM-associated term is labeled in red. (**C**, **C’**) Microtubule-associated terms in Group 3. (**D**) Gene names detected in each GO term shown in (**A**). (**E**) Gene names detected in each GO term shown in (**B**). (**F**) Gene names detected in GO term shown in (**C**). (**G**, **H**) Gene names detected in cell–cell adhesion and GO terms in Group 11. Cell adhesion-related terms are labeled in blue. (**I**) Gene names detected in the cell adhesion-related GO term shown in Group 4. (**J**) Gene name detected in the cell adhesion-related GO term shown in Group 2 and 3.

**Figure EV3:**
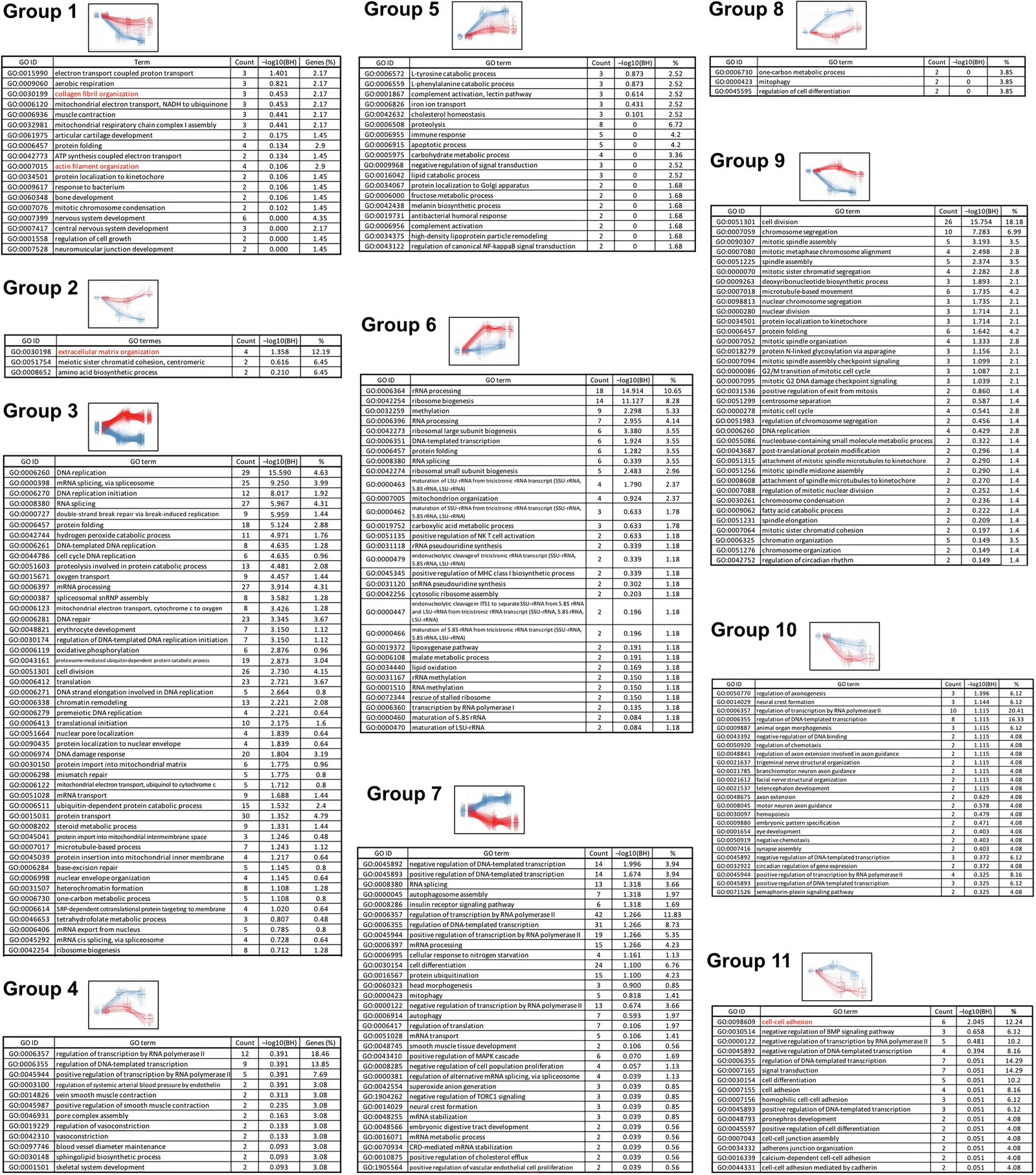
Related to Figs. 1 and 2. GO Biological Process (BP) analysis for each gene expression group shown in Fig. 1D. Genes are grouped based on their temporal expression patterns, and GO analysis is performed for the BP category. Tables show selected BP terms for each group, based on statistical ranking and biological relevance, together with the number and percentage of genes associated with each term, and the corresponding −log_10_(Benjamini-adjusted *P* value). Representative expression patterns for each group are shown above the tables.

Next, we performed GO analysis to predict cellular events that could occur in the thyroid region of fed and unfed tadpoles. In Group 1 and 2, which include genes upregulated under fed conditions and downregulated under unfed conditions, genes related to extracellular matrix organization (GO:0030198), collagen fibril organization (GO:0030199), and actin filament and microtubule organization (GO:0007015, GO:0007017, GO:0007018) were represented (Fig. 2A–C). These GO terms suggest that feeding may promote ECM and cytoskeletal organization (Fig. EV3). Genes associated with these GO terms are shown in Fig. 2D–F. Among ECM-related genes, several collagens are listed in Group 2, and most of them are cartilage-enriched collagens suggesting surrounding lower jaw cartilage may mainly secrete these collagens (Fig. 2B,E). Still, it is notable that collagen, actin cytoskeleton, and microtubule cytoskeleton respond to feeding and may increase their organization or assembly.

**Figure EV4:**
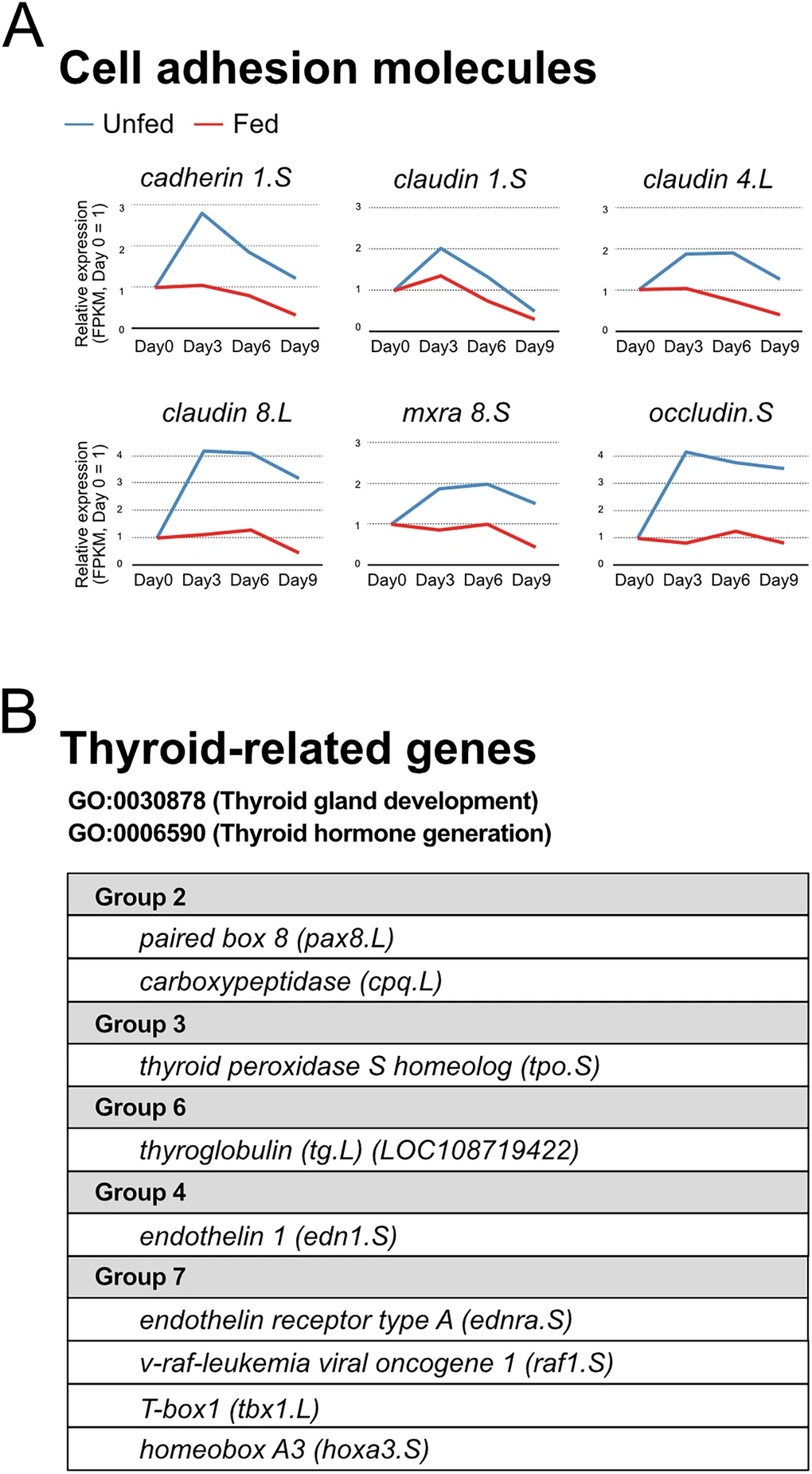
Related to Figs. 1 and 2. (**A**) Additional graphs are provided for the cell adhesion molecules, showing temporal changes in gene expression as relative expression levels (FPKM, Day 0 = 1). (**B**) Thyroid-related genes and their corresponding groups are summarized.

In the Group 4 and 11, which include genes downregulated in the fed and upregulated in the unfed, cell adhesion molecules were identified (Fig. 2H,I). E-cadherin (*cadherin 1: cdh1*) and *claudin 4* are included in the list (Fig. 2I), and their expression levels remained relatively low from Day 0 to Day 9 in the fed group. When examining the temporal expression patterns of cell adhesion molecules using fragments per kilobase of transcript per million mapped reads (FPKM) values from sample 1, several genes showed higher expression in the unfed condition than in the fed condition, particularly at Days 3 and 6 (Fig. EV4A, cell adhesion molecules). For instance, occludin, another epithelial tight junction component, although not included in the list, showed a similar trend, with higher expression in the unfed condition. These expression dynamics suggest that core epithelial cell–cell adhesion is enhanced when thyroid morphogenesis is suppressed under unfed conditions. Overall, the gene expression profiles raise the possibility that the cells adopt a more motile state, characterized by reduced cell adhesion, enriched cytoskeletal components, and a collagen-rich substrate.

### Cell adhesion molecules accumulate more at cell–cell junctions in the thyroid of unfed tadpoles than in fed tadpoles

**Figure 3:**
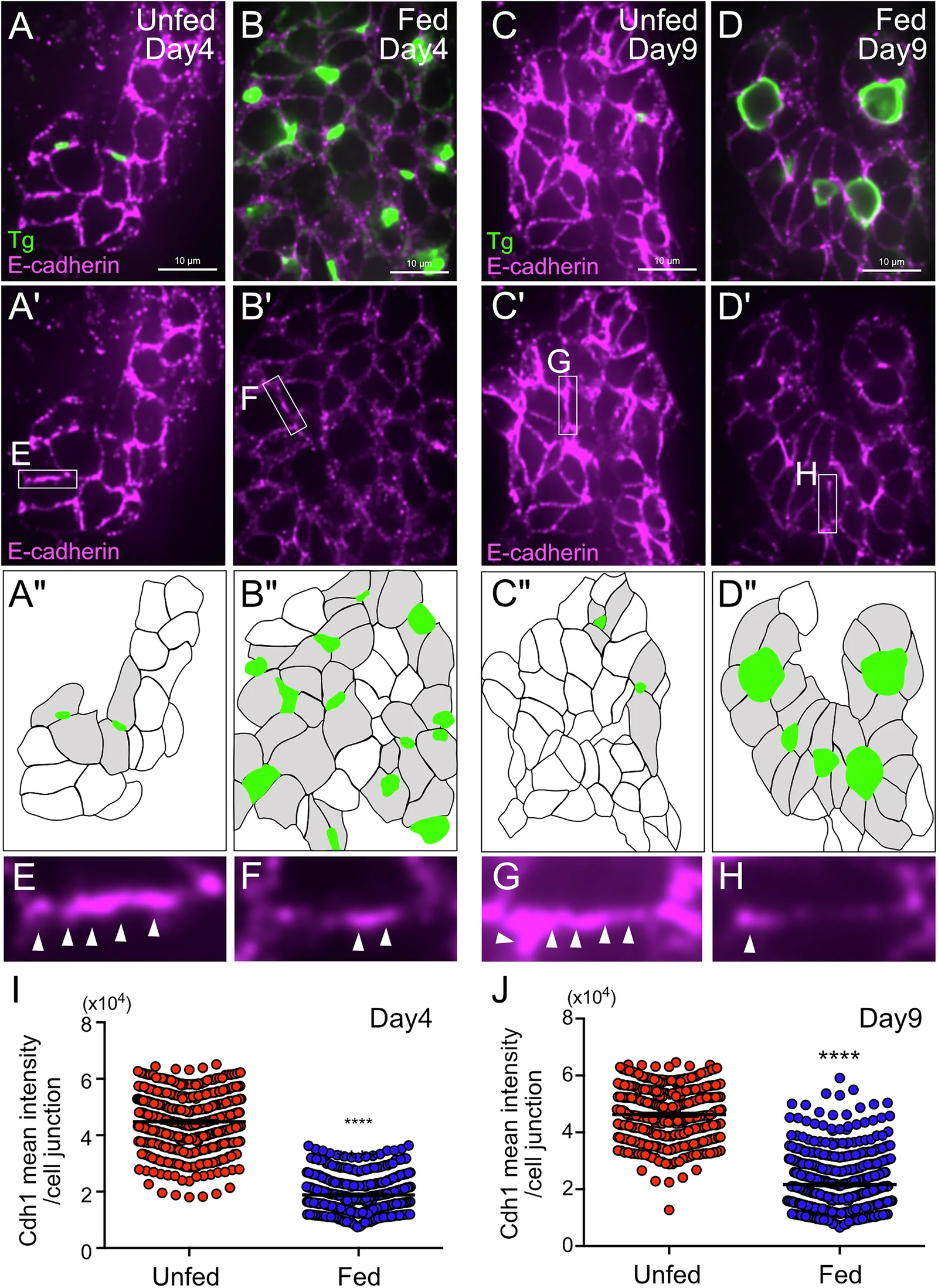
E-cadherin accumulate more at cell–cell junctions in the thyroid of unfed tadpoles than in fed tadpoles. (**A**–**D”**) Immunostaining of the thyroid in unfed (**A**–**A”**, **C**–**C”**) and fed (**B**–**B”**,** D**–**D”**) tadpoles at Day 4 (**A**–**B”**) and Day 8 (**C**–**D”**). Magenta indicates E-cadherin (Cdh1), and green indicates thyroglobulin (Tg). (**A”**, **B”**) Show cell outlines and the location of lumina labeled by Tg. Scale bar = 10 µm. (**E**–**H**) Magnified views of single cell–cell junctions detected in (**A’**–**D’**), respectively. Arrowheads indicate accumulation of E-cadherin along the junction. Scale bar = 2 µm. (**I**, **J**) Quantification of E-cadherin accumulation at cell–cell junctions located 5–10 µm beneath the surface of the thyroid. ****P < 0.0001. Mann–Whitney *U* test. (**I**: Unfed, *n* = 462 cells from 7 tadpoles; Fed, *n* = 455 cells from 7 tadpoles; **J**: Unfed, *n* = 358 cells from 5 tadpoles; Fed, *n *= 553 cells from 8 tadpoles.) Each dot represents one individual cell junction (biological replicate). Experiments were performed using independent clutches. No technical replicates were included. Error bars indicate s.e.m. Source data are available online for this figure.

We then observed E-cadherin protein localization in the thyroid at two time points, Day 4 (Fig. 3A,B”) and Day 9 (Fig. 3C,D”). As we previously reported, thyroid cells of unfed tadpoles show temporal stasis of morphogenesis, maintaining a minimal unit of follicle consisting of two or three cells surrounding a lumen (Fig. 3A–A”,C–C’). Thyroid cells forming these minimal follicular units at Day 4 showed strong E-cadherin localization at the cell–cell junctions (Fig. 3A’), while thyroid cells of fed tadpoles exhibited relatively weak E-cadherin signal (Fig. 3B’). A similar difference in E-cadherin signal between unfed and fed tadpoles was also observed at Day 9, when the fed tadpoles had larger follicles (Fig. 3C–C”,D–D”). We also found that a single cell–cell junction in the thyroid of unfed tadpoles showed uniform accumulation of E-cadherin along the junction (Fig. 3E,G), while the cell junctions in the thyroid of fed tadpoles showed weaker and more polarized localization (Fig. 3F,H). Quantification of the E-cadherin intensity per cell–cell junction showed higher levels in unfed tadpoles than in fed tadpoles at both Day 4 and Day 9 (Fig. 3I,J). These localization data are consistent with the transition of RNA expression presented in Figs. 1 and 2, indicating that feeding reduces E-cadherin localization at cell–cell junctions, whereas starvation enhances its accumulation at these sites in the thyroid.

### Inhibition of E-cadherin promotes thyroid follicle formation and alters cell polarity

**Figure 4:**
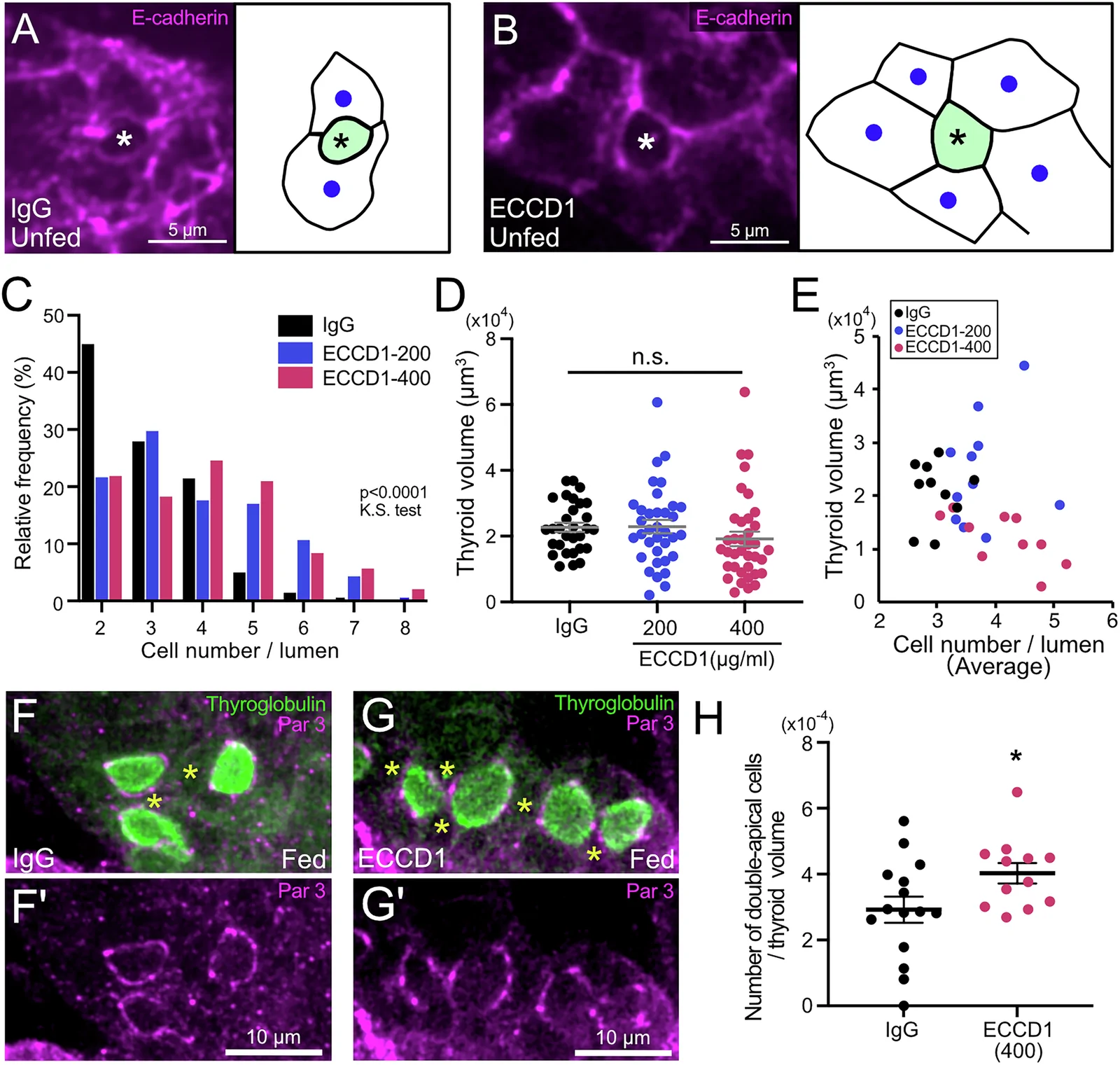
Inhibition of E-cadherin promotes thyroid follicle formation and alters cell polarity. (**A**, **B**) Immunostaining of the thyroid in unfed tadpoles injected with IgG2b (**A**) or ECCD1, an inhibitory antibody against E-cadherin (**B**). Magenta indicates E-cadherin. Asterisk indicates lumen, and blue dots label the cell surrounding lumen. Scale bar = 5 µm. (**C**) Distribution of the number of cells surrounding a single lumen (labeled with blue dots in (**A**, **B**)) in IgG2b-injected control and ECCD1-injected tadpoles (200 µg/ml and 400 µg/ml). Two-sample Kolmogorov–Smirnov tests: IgG vs ECCD1-200, D = 0.2532, *P* < 0.0001; control vs ECCD1-400, D = 0.3271, *P* < 0.0001; Bonferroni corrected. (IgG: *n* = 170 from 9 tadpoles; ECCD1-200 µg/ml: *n* = 173 from 6 tadpoles; ECCD1-400 µg/ml: *n* = 111 from 10 tadpoles). (**D**) Thyroid volume of IgG2b- or ECCD1-injected tadpoles [IgG2b: *n* = 30, ECCD1 (200 µg/ml): *n* = 35, ECCD1 (400 µg/ml) = 37]. One-way ANOVA test. n.s., not significant. Each dot represents one individual thyroid. Experiments were performed using independent clutches. No technical replicates were included. Error bars indicate s.e.m. (**E**) Correlation between the thyroid volume and the average of cell number surrounding a lumen in each thyroid. Each dot represents one individual thyroid. Experiments were performed using independent clutches. (**F**–**G’**) Immunostaining of the thyroid in fed tadpoles injected with IgG2b (**F**) or ECCD1 (**G**). Magenta indicates Par3, and green indicates thyroglobulin. Asterisks mark cells facing two lumina. (**H**) Quantification of the number of cells attached multiple lumina in each thyroid. *P = 0.0469, Mann–Whitney *U* test. (IgG: *n* = 15 thyroids from 8 tadpoles; ECCD1: *n* = 12 thyroids from 7 tadpoles.) Each dot represents one individual cell. Experiments were performed using independent clutches. Error bars indicate s.e.m. Source data are available online for this figure.

Next, the E-cadherin antibody ECCD1, which is a known inhibitory antibody against E-cadherin (Nomura et al, 1986; Ogou et al, 1983; Shigetomi et al, 2018), was injected into the thyroid region of unfed tadpoles to evaluate the significance of E-cadherin accumulation at cell–cell junctions. The tadpoles were injected daily for 5 days without feeding, and then fixed and stained with E-cadherin antibodies. The whole thyroid gland was imaged using Z-stacks covering the full depth of the organ. Imaging revealed that the ECCD1-injected tadpoles exhibited relatively larger lumina in the thyroid compared with the control antibody (IgG2b)-injected tadpoles (Fig. 4A,B). To further assess this enlargement, we quantified follicle size by counting the number of cells per lumen (blue dots in Fig. 4A,B). The ECCD1-injected tadpoles showed significantly larger follicles, with most follicles containing more than four cells (Fig. 4C). Notably, thyroid volume did not differ significantly between ECCD1-injected and control tadpoles (Fig. 4D), and there was no clear correlation between thyroid volume and follicle size (Fig. 4E). These results suggest that the observed follicle enlargement was not due to accelerated growth of the thyroid gland but rather reflects changes in epithelial organization.

We previously found that thyroid cells frequently contact multiple lumina during follicle formation following feeding (Takagishi et al, 2022). This observation raises the possibility that these cells possess multi-axial polarity, defined by the presence of multiple apico–basal axes within a single cell, similar to that observed in liver cells. Although such cells are also observed in the thyroid of unfed tadpoles, they become more frequent and prominent as tadpoles grow after feeding begins. To examine whether thyroid cells develop multi-apical polarity, we observed the localization of Par3, an apical marker of epithelial cells. In fed tadpoles, Par3 consistently localized to the lumen side of thyroid follicles, even in cells with multiple lumina (Fig. 4F,F’, asterisk), supporting the presence of multiple apical domains. ECCD1-injected tadpoles also showed Par3 accumulation at the luminal side comparable to controls (Fig. 4G,G’). Interestingly, ECCD1 injection increased the number of multi-apical cells (Fig. 4H). These results suggest that multi-apical polarity is a normal feature of thyroid morphogenesis, and that reduced cell adhesion following feeding promotes this polarity mode, potentially leading to follicle fusion and enlargement.

### Glycolysis reduces E-cadherin enrichment at cell–cell junctions and enlarges follicles in the thyroid

**Figure 5:**
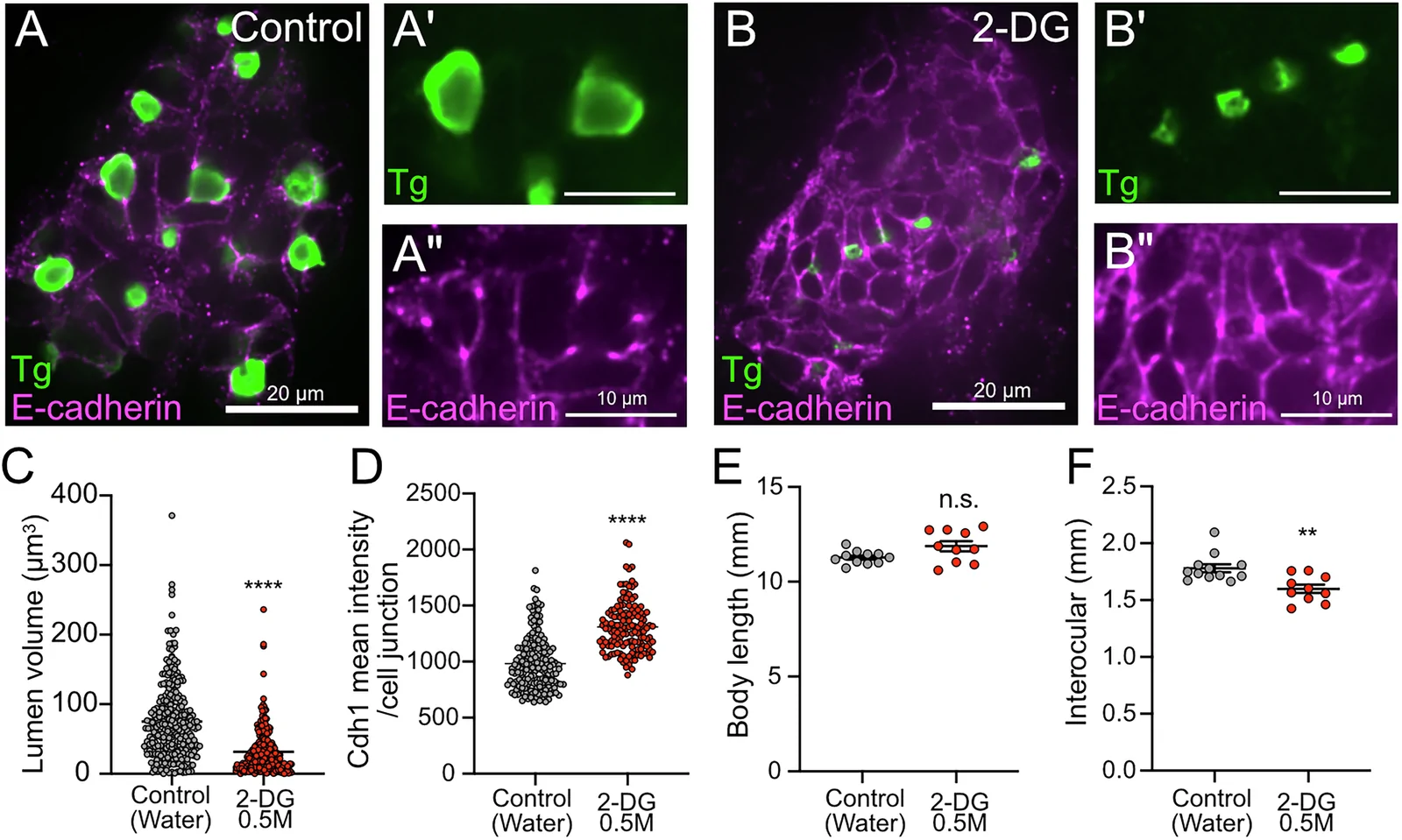
Glycolysis reduces E-cadherin enrichment at cell–cell junctions and enlarges follicles in the thyroid. (**A**–**B”**) Immunostaining of the thyroid in fed tadpoles injected with water (**A**–**A”**) or 2-DG, a glucose analog that suppresses glycolysis (**B**–**B”**). Magenta indicates E-cadherin, and green indicates thyroglobulin (Tg). Scale bars = 20 µm in (**A**, **B**); 10 µm in (**A’**, **A”**, **B’**, **B”**). (**C**) Quantification of lumen volume of each follicle in the thyroid. ****P < 0.0001, Mann–Whitney *U* test. (Control: *n* = 278 lumina from 5 tadpoles; 2-DG: *n* = 241 lumina from 6 tadpoles). Each dot represents one individual lumen. Experiments were performed using independent clutches. Error bars indicate s.e.m. (**D**) Quantification of E-cadherin accumulation at cell–cell junctions located 10–15 µm beneath the thyroid surface. ****P < 0.0001, Mann–Whitney *U* test. (Control: *n* = 183 junctions from 5 tadpoles; 2DG: *n* = 140 junctions from 5 tadpoles). Each dot represents one individual cell junction. Experiments were performed using independent clutches. Error bars indicate s.e.m. (**E**, **F**) Body size of water- or 2-DG–injected tadpoles. Body length shows no significant differences (**E**), while interocular distance is shorter in 2-DG–injected tadpoles (**F**). **P = 0.0026, Mann–Whitney *U* test. (Control: *n* = 11; 2-DG: *n* = 10.) Each dot represents one individual tadpole. Experiments were performed using independent clutches. Error bars indicate s.e.m. Source data are available online for this figure.

To explore whether specific nutrient intake affects E-cadherin levels, we examined the list of genes associated with metabolic pathways in Groups 1, 3, 6, and 9 (Figs. 1 and EV2). The number of genes related to major metabolic pathways—including glycolysis, amino acid biosynthesis, and fatty acid degradation and elongation—was approximately equal, with no particular pathway appeared to be predominant. However, both lactate dehydrogenase (LDH; ldhb.L in Group 3) and glyceraldehyde-3-phosphate dehydrogenase (GAPDH; also in Group 3) were highly upregulated, prompting us to investigate the potential involvement of glycolysis in this process. To inhibit glycolysis, we injected the glucose analog 2-deoxyglucose (2-DG) into fed tadpoles from Day 0 to Day 4, and then the tadpoles were fixed on Day 5. Thyroid tissues were then stained using thyroglobulin and E-cadherin antibodies (Fig. 5A,B).

Quantification of lumen volume revealed that 2-DG–injected tadpoles exhibited smaller lumen volume compared to controls (Fig. 5A’,B’,C). Additionally, E-cadherin showed higher intensity at cell–cell junctions in the 2-DG group, suggesting increased accumulation (Fig. 5A”,B”,D). Interestingly, body length did not differ significantly (Fig. 5E), whereas the interocular distance, another indicator of tadpole body size, was shorter in the 2-DG–injected tadpoles (Fig. 5F). These results suggest that glycolysis contributes to reduced E-cadherin localization and lumen expansion in the thyroid, and may also influence morphogenesis in anterior regions of the body.

### Double-apical cells increase following glucose-enriched feeding or cell adhesion inhibition

**Figure 6:**
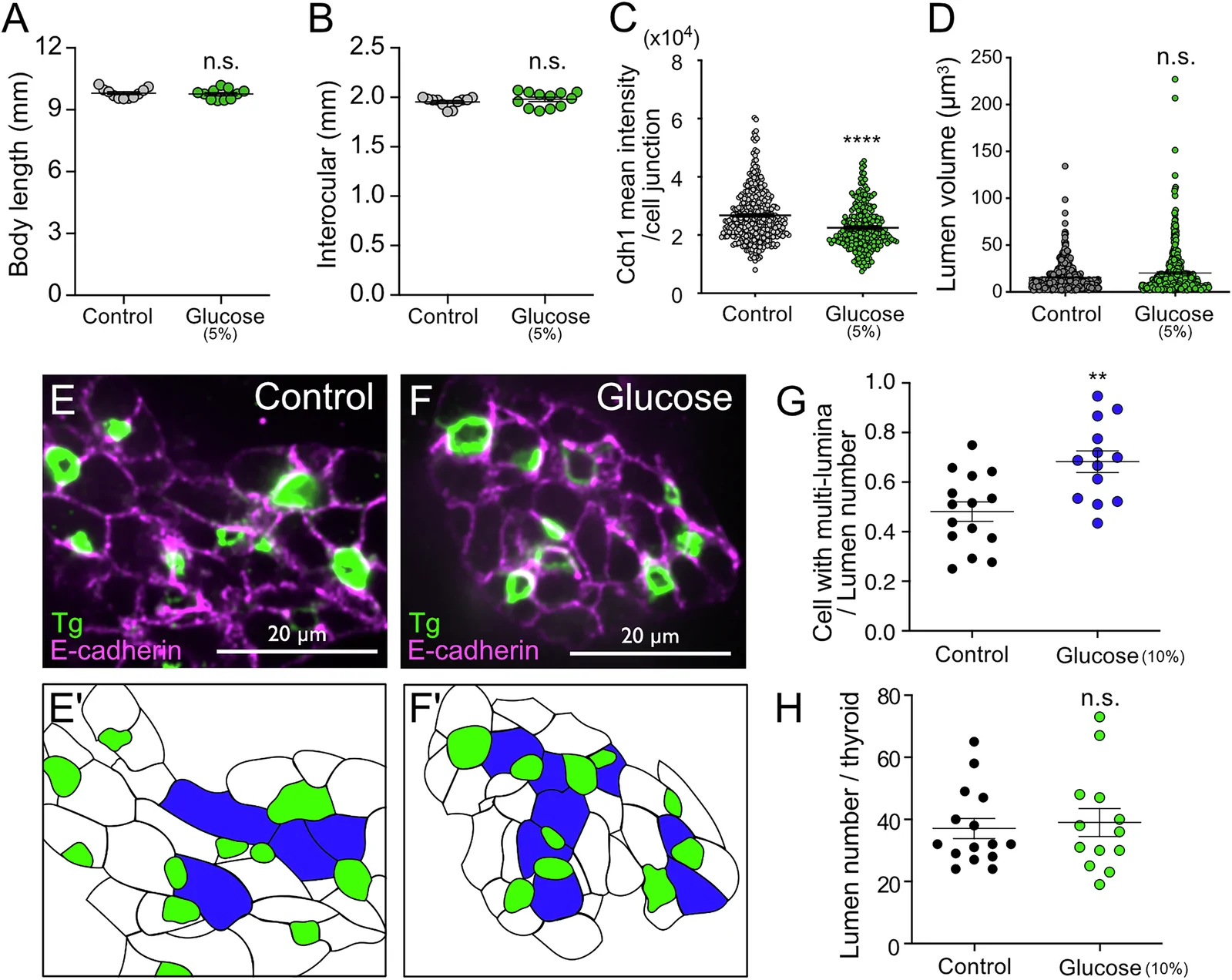
Double-apical cells increase following glucose-enriched feeding or cell adhesion inhibition. (**A**, **B**) Body length (**A**) and interocular distance (**B**) of tadpoles fed with control or glucose-enriched gel for 10 days. (Control: *n* = 11; Glucose: *n *= 12.) Each dot represents one individual tadpole. Experiments were performed using independent clutches. Error bars indicate s.e.m. n.s.: not significant (Mann–Whitney *U* test). (**C**) Quantification of E-cadherin accumulation at cell–cell junctions located 5–10 µm beneath the thyroid surface. ****P < 0.0001, Mann–Whitney *U* test. (Control: *n* = 361 junctions from 9 tadpoles; Glucose: *n* = 241 junctions from 6 tadpoles.) Each dot represents one individual cell junction. Experiments were performed using independent clutches. (**D**) Quantification of lumen volume of thyroid follicles after 14 days of control gel or glucose-enriched gel feeding. n.s., not significant. (Control: *n* = 435 lumina from 8 tadpoles; Glucose: *n* = 404 lumina from 6 tadpoles.) Each dot represents one individual lumen. Experiments were performed using independent clutches. Error bars indicate s.e.m. n.s.: not significant (Mann–Whitney *U* test) *P = 0.0336 (Kolmogorov–Smirnov test). (**E**–**F’**) Immunostaining of the thyroid in tadpoles fed with normal agarose gel (**E**, **E’**) or agarose gel containing 10% glucose for 14 days (**F**, **F’**). Blue-colored cells in (**E’**, **F’**) indicate cells facing multiple lumina. (**G**, **H**) Quantification of the ratio of double-luminal cells in control or glucose-enriched gel feeding. The number of cells contacting multiple lumina per lumen is increased in the glucose-enriched group (**G**), while the number of lumina per thyroid shows no significant differences (**H**). **P = 0.0037, Mann–Whitney *U* test (Control: *n* = 15, Glucose: *n* = 13.) Experiments were performed using independent clutches. Error bars indicate s.e.m. Source data are available online for this figure.

Given that glycolysis regulates cadherin levels in the thyroid, we hypothesized that glucose-enriched food alone may be sufficient to elicit a cellular response. To test this, we prepared glucose-enriched food using agarose gel and fed it to the tadpoles for 10–14 days. Tadpoles fed agarose gel with or without glucose did not increase their body size (Fig. 6A,B), similar to unfed tadpoles (Takagishi et al, 2022). Agarose-based gel alone was expected to provide limited nutritional value because it consists primarily of galactose-derived polysaccharides, which are less effective in supporting glycolytic metabolism (Bustamante and Pedersen, 1977; Weinberg et al, 2010). Nevertheless, the tadpoles consumed the gel-based food, as evidenced by the presence of gel in their cloacal excretions.

Using this artificial diet, we examined the specific effects of glucose supplementation. Thyroid tissues in the glucose-fed group exhibited lower levels of E-cadherin compared to the control group, as confirmed by quantitative analysis of E-cadherin intensity at cell–cell junctions (Fig. 6C). Although the lumen volume was slightly increased in the glucose group, the difference was not substantial compared to the control (Fig. 6D). While the extent of E-cadherin reduction was insufficient to promote robust thyroid follicle enlargement, these findings suggest that dietary glucose alone can reduce E-cadherin levels.

Lastly, we found that the glucose-enriched food increased the number of cells exhibiting multiple apical domains (Fig. 6E,F’). After feeding tadpoles with agarose gel containing 10% glucose, the number of cells with multiple lumina per thyroid was higher than in tadpoles fed agarose gel without glucose (Fig. 6G), while the total number of lumina per thyroid remained unchanged (Fig. 6H). Although glucose-enriched gel was sufficient to elicit changes in thyroid cell properties, it was insufficient to promote body growth, suggesting that additional nutrients are required for body size expansion. These results indicate that the emergence of multi-apical cell polarity occurs downstream of glycolysis activation and reduced cell adhesion, independently of systemic body growth.

## Discussion

Coordinating developmental processes with nutrient availability is critical for organismal survival, yet the mechanisms linking feeding to organ-specific morphogenesis remain poorly understood. In this study, we investigate how feeding influences thyroid development in *Xenopus laevis* tadpoles, building on our previous finding that thyroid morphogenesis is initiated only after feeding. We find that cell adhesion is reduced downstream of glycolysis-associated responses following feeding, thereby promoting thyroid morphogenesis. Although whether this effect is mediated directly by nutrient-derived signals or indirectly via systemic changes remains to be clarified, our results highlight nutritional control of morphogenesis via modulation of cell adhesion dynamics.

The link between glycolytic activity and cell adhesion, well established in cell biology and cultured cell systems, appears to extend to in vivo organogenesis. Our data reveal that nutrient ingestion, likely involving glycolytic activity, modulates cell adhesion levels and localization and promotes thyroid follicle morphogenesis. Previous studies in cultured epithelial and cancer cells have shown that cell adhesion is responsive to nutritional conditions. In these systems, serum starvation has been reported to increase E-cadherin expression via c-Src activation (Dong et al, 2014), and CaMKⅡ-dependent signaling modulates epithelial barrier integrity under nutrient stress (Shiomi et al, 2015). Moreover, adherens junction components such as vinculin are known to associate with cytoskeletal components, potentially contributing to the mechanical stability of cell–cell adhesion upon serum starvation (Hazan et al, 1997). Interestingly, E-cadherin-mediated adhesion can in turn regulate metabolism via mechanotransduction pathways (Bays et al, 2017). In vivo, glycolysis has been linked to adhesive state during collective cell migration, with glucose metabolism contributing to epithelial–mesenchymal transitions in developing tissues in early chick embryos (Bhattacharya et al, 2020). These findings suggest that the relationship between glycolysis and cell adhesion is conserved. Our findings support and expand this concept by showing that nutrient ingestion can regulate cell adhesion in vivo, thereby contributing to organ morphogenesis.

Cell polarity is another important target of glycolysis in our context. While apical-basal cell polarity is often considered stable in epithelial tissues, recent studies suggest that it can be dynamically regulated, exhibiting multi-axial polarity (Overeem et al, 2015; Lu et al, 2025). Further, studies in cancer cells suggest a possible linkage between apical-basal cell polarity and glycolytic activity (Al-Masri et al, 2021). In our study, feeding-induced glycolytic activity led not only to reduction in cell adhesion, but also to a marked increase in thyroid cells exhibiting two apical surfaces, resembling the architecture reported in luminal epithelial cell culture systems. Notably, body size remained unchanged under glucose supplementation, indicating that the observed changes in thyroid cell adhesion and cell polarity are not secondary to systemic growth, but rather reflect direct metabolic regulation at the organ level. Experimental disruption of cell adhesion similarly resulted in an increased number of double-apical cells, suggesting that weakened adhesive contacts may facilitate this shift in polarity. These findings suggest that glycolysis modulates cell polarity at least in part by altering cell–cell adhesion, and that the emergence of multi-apical polarity represents a transitional state favorable for morphogenetic progression. Given that changes in cell polarity accompany follicle expansion, we propose that glycolysis-driven modulation of adhesion and polarity together establish a condition conducive to organ remodeling.

Importantly, despite the increase in multi-apical cells upon increased glycolytic activity, apical polarity itself remains preserved. Consistent with this, Par3 is localized apically at each lumen-facing surface. This suggests that cell polarity is preserved and reorganized, rather than disrupted. In contrast, in cancer cells, loss or mislocalization of Par3 is often associated with polarity breakdown and increased tumorigenicity in a context-dependent manner (Iden et al, 2012; Xue et al, 2013; McCaffrey et al, 2012). Similarly, in MDCK cyst models, Par3 knockdown leads to defective apical domain formation and results in multiple lumina due to polarity failure (Horikoshi et al, 2009). By contrast, in thyroid morphogenesis, the maintained apical localization of Par3 supports the idea that the multi-apical state reflects a regulated remodeling process. Notably, the PAR complex has also been shown to suppress epithelial–mesenchymal transition (EMT) through SNAI1 degradation (Jung et al, 2019), and the preserved cell polarity we observe may contribute to limiting full EMT progression in this context. Although upregulation of ECM and cytoskeletal genes in our RNA-seq analyses may suggest partial EMT, whether cell polarity actively constrains this process remains to be determined.

Although our main analysis focused on thyroid morphogenesis following feeding, a closer look at our RNA-seq data revealed several metabolic pathways that may provide insight into how unfed tadpoles maintain viability without developmental progression. While we did not explore these pathways in detail in this study, transcripts associated with lipid catabolism, as well as sugar- and amino-acid–related pathways, including fructose metabolism, tyrosine catabolism (Nazemi et al, 2024), and phenylalanine catabolism, were also detected under unfed conditions (Group 5 in Figs. 1 and EV3). These signatures may reflect metabolic adaptations that support survival during conditions in which organogenesis is suppressed. Although these pathways were not significantly enriched in our analysis, their detection raise the possibility that developmental arrest in unfed tadpoles involves active metabolic regulation, rather than simply reflecting passive nutrient shortage.

This study has several limitations that should be addressed in future work. First, it is not yet clear whether glycolysis directly regulates cell adhesion and polarity within thyroid cells or acts indirectly through systemic mechanisms. Despite accumulating evidence that glycolytic activity can influence cell adhesion, as discussed above, the extent to which the effects observed here reflect cell-autonomous glycolysis within thyroid cells or systemic metabolic signals remains unresolved. One possible systemic mechanism is endocrine signaling, in which nutrient ingestion triggers the release of circulating factors that influence thyroid morphogenesis. In this context, we explored the potential involvement of the gastrointestinal hormone glucose-dependent insulinotropic polypeptide (GIP), which we previously reported as a candidate mediator of feeding-dependent thyroid morphogenesis, in the regulation of cell adhesion in the thyroid. However, our exploratory observation did not reveal a consistent relationship between GIP signaling and E-cadherin levels at cell–cell junctions in the thyroid. Given that GIP alone is insufficient to sustain thyroid follicle expansion in our earlier study (Takagishi et al, 2022), we consider that GIP signaling may not fully account for the changes in cell adhesion during thyroid morphogenesis. Future work will need to determine whether the link between metabolism, cell adhesion, and cell polarity is specific to the thyroid or reflects a more generalizable mechanism across multiple organs. In addition, whether other nutrient-derived signals, including amino acids and fatty acids, also participate in regulating cell adhesion and polarity remains to be investigated. Further studies will be required to clarify how systemic metabolic signals shape local cellular properties underlying collective cell behaviors, particularly through metabolic analyses aimed at defining unfed- and fed-specific metabolic states.

Our findings demonstrate that nutritional status actively shapes cellular properties and tissue organization during thyroid morphogenesis. By linking glycolytic activity to dynamic changes in cell adhesion and polarity in vivo, this study provides insight into how environmental inputs can regulate cellular mechanisms underlying organ morphogenesis. More broadly, these findings raise the possibility that developing organs adjust their morphogenetic programs to environmental conditions, offering a framework for understanding how external cues are integrated into the processes that shape organ form.

## Methods

**Table:** Reagents and tools table

Reagent/resource	Reference or source	Identifier or catalog number
**Experimental models**		
*Xenopus laevis*	Watanabe Zoushoku, Kato-S-Science	N/A
**Antibodies**		
Rabbit anti-thyroglobulin	Dako	A0251, GA509
Mouse anti-E-cadherin	BD Biosciences	610181
Alexa-Fluor 488 goat anti-rabbit IgG (H + L)	Thermo Fisher Scientific	A-11008
Alexa-Fluor 555 goat anti-mouse IgG (H + L)	Thermo Fisher Scientific	A-21422
Mouse anti-Par 3	Developmental Studies Hybridoma Bank (DSHB)	DA8B4
ECCD1	Takara	M107
**Chemicals, enzymes and other reagents**		
Benzyl benzoate	Fujifilm Wako Pure Chemical Corporation	025-01336
Benzyl alcohol	Fujifilm Wako Pure Chemical Corporation	027-07276
MS-222	Sigma-Aldrich	E10521
D-glucose	Yoneyama Yakuhin Kogyo Co., Ltd.	02281
2-Deoxy-D-glucose	Fujifilm Wako Pure Chemical Corporation	040-06481
**Software**		
Fiji (ImageJ)	Open source	https://imagej.net/Fiji
Prism v10	GraphPad Software	https://www.graphpad.com/features
Aivia v9.8	Leica Microsystems	https://www.aivia-software.com/demo
RNAseqChef	Open source (Etoh and Nakao, 2023)	https://kan-e.github.io/RNAseqChef/
DAVID	National Institutes of Health	https://davidbioinformatics.nih.gov/workspace.html
**Other**		
TRIzol LS reagent	Thermo Fisher Scientific	10296010
RNA Clean & Concentrator-5	Zymo Research	R1014

### Methods and protocols

#### RNA-seq analysis

Total RNA was extracted from isolated thyroid and lower jaw cartilage tissues from St. 46 tadpoles, as well as from fed and unfed tadpoles at Day 3, 6, 9 after feeding onset. Duplicate samples were prepared for each time point, except for Day 9, for which only a single sample was obtained due to the limited number of tadpoles. Thyroid regions were dissected using a thin razor and fine forceps. A total of 20–40 pieces per stage were collected and homogenized in TRIzol LS reagent (#10296010; Thermo Fisher Scientific). Total RNA was extracted and purified using the RNA Clean & Concentrator-5 kit (R1014; Zymo Research, Irvine, CA, USA). Purified total RNA was outsourced to Takara Bio Inc. for RNA-seq library preparation and sequencing. Libraries were prepared using SMART-Seq v4 Ultra Low Input RNA Kit (Takara Bio) and sequencing was performed on an Illumina NovaSeq 6000 platform according to the service provider’s protocols.

For data analysis, raw count data were used for divisive clustering analysis with RNAseqChef (version1.1.7) developed by Dr. Kan Etoh (https://kan-e.github.io/RNAseqChef/, https://github.com/Kan-E/RNAseqChef/wiki) (Etoh and Nakao, 2023). The FDR was calculated using the Benjamini–Hochberg (BH) method. The cut-off conditions were set as follows: fold change, 1.5; FDR, 0.1; and base mean, 0. A pair of fold-change cut offs was applied between Day 6 Unfed and Day 6 Fed. The number of most significant genes was set to 3500 out of 11,283 genes. As differences between fed and unfed at Day 3 were often inconsistent, these samples were excluded to better resolve distinct clusters. KEGG pathway and GO term enrichment analyses were performed for each group using DAVID (https://davidbioinformatics.nih.gov/).

#### Immunostaining and antibodies

The fixation and preparation of thyroid tissues for staining was previously described (Takagishi et al, 2022; Shindo et al, 2018). Tadpoles were fixed in 2% trichloroacetic acid at 20–23 °C for 25 min. Fixed tadpoles were washed with 0.3% PBST (PBS + 0.3% Triton x100), and thyroid tissues with lower jaw cartilage were isolated. Isolated tissues were incubated in 10% goat serum for 6 h at 25 °C. Samples were incubated with primary antibodies at 4 °C overnight: rabbit anti-thyroglobulin (1:1000, A0251, RRID: AB_10013723; Dako, Berlin, Germany; or GA509; Dako, Berlin, Germany), mouse anti-E-cadherin (1:300, #610181, RRID: AB_397580; BD Biosciences, Franklin Lakes, NJ, USA), and mouse anti-Par3 (1:50, DA8B4, Developmental Studies Hybridoma Bank, The University of Iowa, USA). After primary antibody incubation, samples were washed with 0.3% PBST and subsequently treated with the following secondary antibodies for 2 h at 25 °C: Alexa-Fluor 488 goat anti-rabbit IgG (H + L) (1:1000, A-11008, RRID: AB_143165; Thermo Fisher Scientific, Waltham, MA, USA) and Alexa-Fluor 555 goat anti-mouse IgG (H + L) (1:300, A-21422, RRID: AB_2535844; Thermo Fisher Scientific).

#### Thyroid imaging

After immunostaining, thyroid tissues were dehydrated in methanol with five solution exchanges to ensure complete dehydration. The dehydrated thyroid tissues were cleared in a 2:1 mixture of benzyl benzoate (#025-01336; Wako) and benzyl alcohol (#027-07276; Wako). Cleared thyroid tissues were then imaged along the *z* axis using the Cell Voyager CV1000 Confocal Scanner Box (Yokogawa Electric, Tokyo, Japan) or Spin10 spinning disk confocal microscopy (Evident, Japan), with z-stacks acquired at intervals of 0.25–0.5 µm.

#### Inhibitory reagents injection

Tadpoles were anesthetized in 0.25% MS-222 (E10521; Sigma-Aldrich, St. Louis, MO, USA), and 2-Deoxy-D-glucose (2-DG, FujiFilm Wako, 040-06481) or ECCD1 (200 µg/ml, 400 µg/ml, Takara, M107) was prepared for injection. Anesthetized tadpoles were placed ventral side up on a moist Kimwipes^®^, and injections were performed from the anterior side targeting the thyroid region. A volume of 20 nl of either 0.5 or 1 M 2-DG solution or ECCD1, was injected subdermally using a glass needle and microinjector ((IM-400, IM-300; Narishige, Tokyo, Japan).

#### Nutrient-restricted food preparation and feeding

Nutrient-restricted food was prepared by making 1% agarose gel in a plastic dish. After the gel had solidified, a 0.5 g block was cut out and crushed. The crashed agarose gel was fed to tadpoles as nutrient-restricted food. For glucose-enriched food, D-glucose (02281; Yoneyama Yakuhin Kogyo Co., Ltd., Osaka, Japan) was dissolved in 1% agarose at a final concentration of 5% or 10% (w/v). After solidification, an equivalent amount of glucose-containing gel was crushed and fed to tadpoles.

#### Image analysis (volume measurements of the lumen)

Lumen volume was detected using a deep learning model in Aivia v9.8 (https://www.aivia-software.com/). Thyroglobulin-stained lumen areas were manually filled using ImageJ v1.52p. The manually filled images were used as labeled training data to train the deep learning model. The trained deep learning model was applied to all raw images. The detected lumen region was analyzed using the 3D object analysis protocol in Aivia to measure the volume of each follicle. No formal blinding was applied. Quantitative image-based analyses were performed using predefined criteria to minimize subjective bias.

#### Quantification and statistical analysis

Statistical analyses were performed using Prism v10 (GraphPad Software, San Diego, CA, USA). The statistical tests for each analysis are described in the figure legends.

## Supplementary information

Peer Review File

Dataset EV1

Source data Fig. 1

Source data Fig. 3

Source data Fig. 4

Source data Fig. 5

Source data Fig. 6

Expanded View Figures

## Data Availability

RNA-seq data have been deposited in the NCBI Sequence Read Archive (SRA) under BioProject accession number PRJNA1427430. The corresponding SRA accession numbers are SRR37344805, SRR37344806, SRR37344807, SRR37344808, SRR37344809, and SRR37344810. The source data of this paper are collected in the following database record: biostudies:S-SCDT-10_1038-S44319-026-00868-4.
