# Supplementary material for: Glycolytic suppression of cell adhesion promotes thyroid morphogenesis in response to nutrition
Source: EMBO Rep. 2026 Jul 20;27(17):5143–61. doi: 10.1038/s44319-026-00868-4 (PMC13554297; doi:10.1038/s44319-026-00868-4)
Supplement: Supplementary file 1 [file 44319_2026_868_MOESM1_ESM.pdf]

EMBO reports

Glycolytic suppression of cell adhesion promotes thyroid morphogenesis in response to nutrition

Asako Shindo, Shiho Nishiguchi, Ayaka Fujiwara, and Kaoru Nakashima

Corresponding author(s): Asako Shindo (shindo.asako.sci@osaka-u.ac.jp)

Review Timeline:

Submission Date:	21st Jul 25
Editorial Decision:	26th Aug 25
Revision Received:	25th Feb 26
Editorial Decision:	2nd Apr 26
Revision Received:	20th Jun 26
Accepted:	25th Jun 26

Editor: Deniz Senyilmaz Tiebe / Esther Schnapp

Transaction Report:

(Note: With the exception of the correction of typographical or spelling errors that could be a source of ambiguity, letters and reports are not edited. Depending on transfer agreements, referee reports obtained elsewhere may or may not be included in this compilation. Referee reports are anonymous unless the Referee chooses to sign their reports.)

1st Editorial Decision 26th Aug 2025

Dear Dr. Shindo,

Thank you for transferring your manuscript to EMBO Reports, which was now seen by two referees, whose reports are copied below.

Referees express interest in the proposed role of nutritional availability in thyroid morphogenesis in Xenopus tadpoles. However, they, especially referee #1, also raise some concerns that need to be addressed to consider publication here.

I find the reports informed and constructive, and believe that addressing the concerns raised will significantly strengthen the manuscript. As the reports are below, and I think all points need to be addressed, I will not detail them here. Please contact me if you have questions or comments regarding the revision for further discussion (also by video chat).

Given these positive recommendations, we would like to invite you to revise your manuscript with the understanding that the referee concerns (as in their reports) must be fully addressed and their suggestions taken on board. Please address all referee concerns in a complete point-by-point response. Acceptance of the manuscript will depend on a positive outcome of a second round of review. It is EMBO reports policy to allow a single round of major experimental revision only and acceptance or rejection of the manuscript will therefore depend on the completeness of your responses included in the next, final version of the manuscript.

We realize that it is difficult to revise to a specific deadline. In the interest of protecting the conceptual advance provided by the work, we recommend a revision within 3 months. Please discuss the revision progress ahead of this time with me if you require more time to complete the revisions, or if you have questions or comments regarding the revision (also by video chat).

IMPORTANT NOTE: we perform an initial quality control of all revised manuscripts before re-review. Your manuscript will FAIL this control and the handling will be DELAYED if the following APPLIES:
1. A data availability section providing access to data deposited in public databases is missing (where applicable).
2. Your manuscript contains statistics and error bars based on n=2. Please use scatter plots in these cases.

You can submit the revision either as a Scientific Report or as a Research Article. For Scientific Reports, the revised manuscript can contain up to 5 main figures and 5 Expanded View figures, and it should not exceed 27000 characters. If the revision leads to a manuscript with more than 5 main figures it will be published as a Research Article. In this case the Results and Discussion section should be separate. If a Scientific Report is submitted, these sections have to be combined. This will help to shorten the manuscript text by eliminating some redundancy that is inevitable when discussing the same experiments twice. In either case, all materials and methods should be included in the main manuscript file.

When submitting your revised manuscript, please carefully review the instructions that follow below. Failure to include requested items will delay the evaluation of your revision.

1) a .docx formatted version of the manuscript text (including legends for main figures, EV figures and tables). Please make sure that the changes are highlighted to be clearly visible.

2) individual production quality figure files as .eps, .tif, .jpg (one file per figure). See https://wol-prod-cdn.literatumonline.com/pb-assets/embo-site/EMBOPress_Figure_Guidelines_061115-1561436025777.pdf for more info on how to prepare your figures.

3) We replaced Supplementary Information with Expanded View (EV) Figures and Tables that are collapsible/expandable online. A maximum of 5 EV Figures can be typeset. EV Figures should be cited as 'Figure EV1, Figure EV2" etc... in the text and their respective legends should be included in the main text after the legends of regular figures.

- For the figures that you do NOT wish to display as Expanded View figures, they should be bundled together with their legends in a single PDF file called *Appendix*, which should start with a short Table of Content. Appendix figures should be referred to in the main text as: "Appendix Figure S1, Appendix Figure S2" etc. See detailed instructions regarding expanded view here: <https://www.embopress.org/page/journal/14693178/authorguide#expandedview>

- Additional Tables/Datasets should be labeled and referred to as Table EV1, Dataset EV1, etc. Legends have to be provided in a separate tab in case of .xls files. Alternatively, the legend can be supplied as a separate text file (README) and zipped together with the Table/Dataset file.

4) a .docx formatted letter INCLUDING the reviewers' reports and your detailed point-by-point responses to their comments. As part of the EMBO publication's Transparent Editorial Process, EMBO reports publishes online a Review Process File (RPF) to accompany accepted manuscripts. This File will be published in conjunction with your paper and will include the referee reports,

your point-by-point response and all pertinent correspondence relating to the manuscript.
https://www.embopress.org/page/journal/14693178/authorguide#transparentprocess
You are able to opt out of this by letting the editorial office know (emboreports@embo.org). If you do opt out, the Review Process File link will point to the following statement: "No Review Process File is available with this article, as the authors have chosen not to make the review process public in this case."

5) a complete author checklist, which you can download from our author guidelines
https://www.embopress.org/page/journal/14693178/authorguide. Please insert information in the checklist that is also reflected in the manuscript. The completed author checklist will also be part of the RPF.

6) Please note that all corresponding authors are required to supply an ORCID ID for their name upon submission of a revised manuscript (<https://orcid.org/>). Please find instructions on how to link your ORCID ID to your account in our manuscript tracking system in our Author guidelines
<https://www.embopress.org/page/journal/14693178/authorguide#authorshipguidelines>

7) Before submitting your revision, primary datasets produced in this study need to be deposited in an appropriate public database (see https://www.embopress.org/page/journal/14693178/authorguide#datadeposition). Please remember to provide a reviewer password if the datasets are not yet public. The accession numbers and database should be listed in a formal "Data Availability" section placed after Materials & Method (see also
https://www.embopress.org/page/journal/14693178/authorguide#datadeposition). Please note that the Data Availability Section is restricted to new primary data that are part of this study. * Note - All links should resolve to a page where the data can be accessed. *
If your study has not produced novel datasets, please mention this fact in the Data Availability Section.

8) At EMBO Press we ask authors to provide source data for the main manuscript figures. You will receive a separate email with instructions for providing source data with your revised manuscript, including how to upload and organize the files.

Additional information on source data and instruction on how to label the files are available:
https://www.embopress.org/page/journal/14693178/authorguide#sourcedata

9) Our journal encourages inclusion of *data citations in the reference list* to directly cite datasets that were re-used and obtained from public databases. Data citations in the article text are distinct from normal bibliographical citations and should directly link to the database records from which the data can be accessed. In the main text, data citations are formatted as follows: "Data ref: Smith et al, 2001" or "Data ref: NCBI Sequence Read Archive PRJNA342805, 2017". In the Reference list, data citations must be labeled with "[DATASET]". A data reference must provide the database name, accession number/identifiers and a resolvable link to the landing page from which the data can be accessed at the end of the reference. Further instructions are available at http://www.embopress.org/page/journal/14693178/authorguide#referencesformat

10) Regarding data quantification (see Figure Legends:
https://www.embopress.org/page/journal/14693178/authorguide#figureformat)

The following points must be specified in each figure legend:

- the name of the statistical test used to generate error bars and P values,

- the number (n) of independent experiments (please specify technical or biological replicates) underlying each data point,

- the nature of the bars and error bars (s.d., s.e.m.),

- If the data are obtained from n Program fragment delivered error ``Can't locate object method "less" via package "than" (perhaps you forgot to load "than"?) at //ejpvfs23/sites23b/embor_www/letters/embor_decision_revise_and_review.txt line 56.' 2, use scatter blots showing the individual data points.

Discussion of statistical methodology can be reported in the materials and methods section, but figure legends should contain a basic description of n, P and the test applied.

- Please also include scale bars in all microscopy images.

11) The journal requires a statement specifying whether or not authors have competing interests (defined as all potential or actual interests that could be perceived to influence the presentation or interpretation of an article). In case of competing interests, this must be specified in your disclosure statement. Further information: https://www.embopress.org/competing-interests

12) Please also note our reference format:
http://www.embopress.org/page/journal/14693178/authorguide#referencesformat

13) All Materials and Methods need to be described in the main text using our 'Structured Methods' format, which is required for all research articles. According to this format, the Methods section includes a Reagents and Tools Table (listing key reagents, experimental models, software and relevant equipment and including their sources and relevant identifiers) followed by a Methods and Protocols section describing the methods using a step-by-step protocol format. The aim is to facilitate adoption of the methodologies across labs. More information on how to adhere to this format as well as a downloadable template (.docx) for the Reagents and Tools Table can be found in our author guidelines:
https://www.embopress.org/page/journal/14693178/authorguide#structuredmethods.

An example of a Method paper with Structured Methods can be found here:
https://www.embopress.org/doi/10.15252/msb.20178071.

We would also welcome the submission of cover suggestions, or motifs to be used by our Graphics Illustrator in designing a cover.

I look forward to seeing a revised version of your manuscript when it is ready. Please let me know if you have questions or comments regarding the revision.

Kind regards,

Deniz Senyilmaz Tiebe

Deniz Senyilmaz Tiebe, PhD
Senior Scientific Editor
EMBO Reports

Referee #1:

This manuscript by Shindo et al investigates the morphogenetic effects in the thyroid associated with nutritional availability in growing Xenopus tadpoles. Starting with a genome-wide investigation of transcriptional changes found in fed versus unfed tadpole thyroid, the authors further focus on changes in E-cadherin expression potentially underlying thyroid morphogenesis. The manuscript is an extension of a previous paper (Takagishi et al., Current Biology 2022).
While the manuscript certainly has interesting data and would be worth publishing, it somewhat lacks body and insights into mechanism that would increase its potential. Interestingly, their previous study mentioned above, provides the experiments that in my opinion should be able to address some of the unknowns and speculations in the present manuscript.

As acknowledged by the authors, a major shortcoming in the manuscript is that the presented experiments do not properly allow to distinguish between direct and indirect effects of nutrient derived signals. The authors also fail to discuss the current findings with the data of their 2022 paper (Takagishi et al.). Hereby it would be interesting to see how injection of Gastric Inhibitory Peptide (GIP) or GIP/GLP-1 receptor antagonist peptide (exendin 3) (both described in Takagishi et al., 2022) affect E-cadherin levels in their experimental setting. Or does the experiment with the glucose analog 2-deoxyglucose (2-DG) disfavor indirect GIP involvement? The local intradermal injection of the 2-DG could in my opinion still exert indirect effects since it would easily diffuse in the circulation. It would in this context be interesting to see whether 2-DG could prevent GIP-dependent lumen-formation.

A technical experiment that should be improved is the control for the injections of the E-cadherin neutralizing ECCD1 antibody. Rather than water, a control antibody should be injected.

Referee #2:

This manuscript follows up on previous studies on the formation of the thyroid gland in Xenopus. It shows that fed and unfed tadoles have substantially different thyroid morphology and apical basal polarity in the follicles. By measuring gene expression they document that adhesion differences are substantially different, and that modulation of adhesion by Cdh1 can be inhibited and promoted thyroid follicle formation. Indeed activation of glycolysis suppresses call adhesion, and the effect is independent of grwoth of the overall tadpole, which links the metabolic activity to regulation of adhesion. They also observe that promotion of glycolysis and the concomitant reduction of adhesion results in multi luminal structures, with different polarity from the unfed follicle, while reduction of glycolysis has the opposite effect.
The data are clear,and the experiments well designed to show these findings. The interesting findings on the effect of metaboilism on adhesion and resulting structural maturation will be very interesting to readers of EMBO Journal

Dear Editor and Reviewers,

Thank you very much for the time and effort you have devoted to evaluating our manuscript. We sincerely apologize for the delay in submitting the revision and greatly appreciate your patience. The revision required additional time because we carefully re-examined several datasets, including results that were complex or did not show a single consistent trend, to ensure that our conclusions are presented accurately and transparently.
In the revised version, we have carefully addressed all points raised by the reviewers, and we provide below a detailed, point-by-point response describing the corresponding changes. All modifications in the revised manuscript are indicated in red text.

In the responses below, reviewer comments are shown in blue, and our responses are provided in black text for clarity.

Referee #1:
This manuscript by Shindo et al investigates the morphogenetic effects in the thyroid associated with nutritional availability in growing Xenopus tadpoles. Starting with a genome-wide investigation of transcriptional changes found in fed versus unfed tadpole thyroid, the authors further focus on changes in E-cadherin expression potentially underlying thyroid morphogenesis. The manuscript is an extension of a previous paper (Takagishi et al., Current Biology 2022).
While the manuscript certainly has interesting data and would be worth publishing, it somewhat lacks body and insights into mechanism that would increase its potential. Interestingly, their previous study mentioned above, provides the experiments that in my opinion should be able to address some of the unknowns and speculations in the present manuscript.

As acknowledged by the authors, a major shortcoming in the manuscript is that the presented experiments do not properly allow to distinguish between direct and indirect effects of nutrient derived signals. The authors also fail to discuss the current findings with the data of their 2022 paper (Takagishi et al.). Hereby it would be interesting to see how injection of Gastric Inhibitory Peptide (GIP) or GIP/GLP-1 receptor antagonist peptide (exendin 3) (both described in Takagishi et al., 2022) affect E-cadherin levels in their experimental setting. Or does the experiment with the glucose analog 2-deoxyglucose (2-DG) disfavor indirect GIP involvement? The local intradermal injection of the 2-DG could in my opinion still exert indirect effects since it would easily diffuse in the circulation. It would in this context be interesting to see whether 2-DG could prevent GIP-dependent lumen-formation.
A technical experiment that should be improved is the control for the injections of the E-cadherin neutralizing ECCD1 antibody. Rather than water, a control antibody should be injected.

Response:
Thank you for the valuable suggestions. We appreciate the reviewer's reference to our previous work. As suggested, we performed the following three experiments to investigate the link between the GI hormone axis and the expression of a cell adhesion molecule in the thyroid.
(1) E-cadherin immunostaining after GIP injection.
(2) E-cadherin immunostaining after GIP/GLP-1 receptor antagonist peptide in fed condition.
(3) Simultaneous injections of 2-DG and GIP into unfed tadpoles.

We also performed additional ECCD1 experiments using an appropriate control (experiment 4).

For experiments (1)–(3), our analyses overall did not show a clear relationship between GIP signaling and cell adhesion in the thyroid. The experimental outcomes did not reveal a consistent pattern linking GIP to E-cadherin levels, and attempts to examine GIP function together with glycolysis raised broader mechanistic questions that would require substantially different, omics-level approaches beyond the central scope of the present manuscript. We have therefore kept these new aspects for future study and have kept the discussion of GIP concise in the revised manuscript based on the considerations outlined below.

The detailed experimental results that led to this conclusion are described below.

(1) E-cadherin immunostaining after GIP injection:

If the reduction of cell adhesion in the thyroid after feeding were mediated by GIP secretion from the intestine, GIP-injected tadpoles would be expected to show reduced E-cadherin levels. However, we did not observe a consistent or directional relationship between GIP signaling and E-cadherin levels (see graphs below). These conclusions are based on five newly performed experiments as well as re-quantification of our previously published data.

Figure for referee with unpublished data has been removed upon request by the authors.

Our previous work showed that GIP alone is not sufficient to sustain or complete follicle enlargement in the thyroid. One possible explanation of the present results is that GIP may contribute to specific aspects of thyroid morphogenesis, such as lumen merging, but it does not reliably induce the reduction in E-cadherin levels that may be required for continuous follicle enlargement process. Please refer to experiment (3) for further discussion.

(2) E-cadherin immunostaining after GIP/GLP-1 receptor antagonist peptide in fed condition:

As the reviewer pointed out, if feeding- or glycolysis-induced reduction of E-cadherin is mediated by GIP, then inhibition of GIP should also induce an increase in E-cadherin in the fed condition. However, as well as the experiment of GIP injection above, the results are not consistent. The mean E-cadherin intensity was comparable to the fed or sometimes lower or slightly higher. These results are derived from three independent experiments together with re-quantification of datasets from our published studies (See the graphs below).

Figure for referee with unpublished data has been removed upon request by the authors.

Together with the results of (1), GIP may not directly alter E-cadherin levels. E-cadherin appears to decrease in a certain level upon feeding regardless of GIP inhibition, indicating that blocking GIP signaling does not significantly alter this change. This raises the possibility that feeding-derived nutrient signals and GIP signaling contribute to follicle enlargement through distinct pathways. However, the present results do not allow us to conclude this clearly, nor to distinguish whether feeding regulates cell adhesion in the thyroid directly or indirectly. While these experiments provided useful additional insight, they did not substantially change the overall interpretation of our study. Therefore, we have described this possibility only briefly in the Discussion section.

(3) Simultaneous injections of 2-DG and GIP into unfed tadpoles:

We believe that this experiment raised mechanistic questions that extend beyond the central focus of the present manuscript. We performed simultaneous injections of 2-DG and GIP into unfed tadpoles as the reviewer requested. Interestingly, GIP and 2-DG did not exhibit the anticipated opposing effects on E-cadherin; instead, we observed an unexpected enlargement of the thyroid and follicular lumen (see below).

Figure for referee with unpublished data has been removed upon request by the authors.

We currently do not have a definitive explanation for this result. Consistent with our data in the present manuscript showing that 2-DG tends to increase cell adhesion, the 2-DG-injected groups show higher E-cadherin intensity. However, co-injection of 2-DG and GIP increased thyroid and lumen size without reducing E-cadherin levels. This observation is compatible with the results of (1) and (2), which indicate that GIP and E-cadherin levels are not linearly correlated.

Nevertheless, the situation appears to be more complex, as we also have found that the retention of embryonic nutrients (yolk) may influence the effect of GIP and 2-DG. When developmental speed was accelerated by increasing temperature, resulting in possible greater retention of embryonic nutrients, the combined treatment with 2-DG and GIP no longer increased thyroid and lumen volume. These complex data raise the possibility that nutrient-deprived tadpoles engage a distinct regulatory mechanism that modulates glycolysis and morphogenesis to conserve energy, and that 2-DG may interfere with this system. This also highlights another important question: how tadpoles tolerate starvation conditions.

Although the data of (1)–(3) were variable, they did not yield a clear conclusion directly relevant to the central scope of the present study. Furthermore, these observations currently lack a clear mechanistic framework and would remain largely descriptive; therefore, we have chosen not to incorporate them into the Results. However, as the reviewer suggested, it is necessary to discuss the current findings together with the data from our previous paper. We have now added a discussion addressing these observations and outlining potential directions for future mechanistic investigations (page 8, paragraph starting with “This study has several limitations that should be addressed….”).

We hope that the reviewer will find our responses and additional experiments satisfactory. We sincerely thank the reviewer for encouraging these analyses, which have provided valuable insights and will help guide our future studies.

(4) ECCD1 injection experiments using IgG2b as the appropriate control:

We reperformed the ECCD1 injection experiments using IgG2b as a control. The overall conclusions remain unchanged from our original manuscript. However, the anti-thyroglobulin antibody that we previously used as a lumen marker is no longer available, and the new replacement antibody did not stain the lumen as clearly as before, particularly in the thyroid under unfed conditions. Therefore, we quantified the number of cells per lumen using only E-cadherin staining (revised Fig. 4A, B). The E-cadherin antibody still labels cell-cell borders after ECCD1 injection, and the lumen can be outlined by apically accumulated E-cadherin, allowing us to count the number of cells surrounding each lumen as in the original manuscript. The updated data are now presented in Fig. 4C.

Another revision to Figure 4 is the inclusion of thyroid volume measurements. In the original version, we presented body size data to show that ECCD1 injection did not affect overall tadpole growth, however, we consider thyroid volume to be a more relevant parameter for evaluation whether inhibition of E-cadherin influences tissue growth. As expected, thyroid volume did not differ significantly among groups, and no correlation was observed between thyroid volume and the average number of cells per lumen (Fig. 4D, E). These results further support our conclusion. Fig. 4F and 4G have also been replaced with experiments performed using the appropriate IgG2b control.

The main text has been modified accordingly. Please see page 5, the section entitled “Inhibition of E-

cadherin promotes thyroid follicle formation and modifies cell polarity".

Referee #2:

This manuscript follows up on previous studies on the formation of the thyroid gland in Xenopus. It shows that fed and unfed tadpoles have substantially different thyroid morphology and apical basal polarity in the follicles. By measuring gene expression they document that adhesion differences are substantially different, and that modulation of adhesion by Cdh1 can be inhibited and promoted thyroid follicle formation. Indeed activation of glycolysis suppresses call adhesion, and the effect is independent of growth of the overall tadpole, which links the metabolic activity to regulation of adhesion. They also observe that promotion of glycolysis and the concomitant reduction of adhesion results in multi luminal structures, with different polarity from the unfed follicle, while reduction of glycolysis has the opposite effect.

The data are clear, and the experiments well designed to show these findings. The interesting findings on the effect of metabolism on adhesion and resulting structural maturation will be very interesting to readers of EMBO Journal.

We appreciate the reviewer's positive assessment and are pleased that no further revisions were requested for this point.

Additional minor revisions made to correct errors and improve clarity are summarized below. These changes were not directly requested by the reviewers but were implemented to improve readability.

・Figure EV3: Individual graphs showing temporal gene expression changes were added. These panels present the expression dynamics of individual cell adhesion molecules that were missing from the original manuscript, thereby clarifying the focus of the study (page 3, the paragraph starting with "In the Group 9, involving genes…").

・Figure 6: Overlapping panels were removed. To simplify the figure, images of control and glucose-feeding samples are now shown only in Fig. 5E and 5F. In the original figure, four images were included in Fig 6C–D, 6G–H; however, these paired images represented the same context and did not provide additional information.

・"E-cadherin" is now used consistently throughout the manuscript and figures, except when referring to the gene name cdh1, to improve clarity and avoid confusion between gene and protein nomenclature.

Dear Dr. Shindo,

Thank you for the submission of your revised manuscript. We have now received the enclosed report from referee 1 and I am happy to say that this referee supports the publication of your study now. Only a few editorial requests will need to be addressed before we can proceed with the official acceptance of your manuscript:

- Please correct the conflict of interest subheading to "Disclosure and Competing Interests Statement".

- The author credits need to be removed from the ms file. All credits need to be entered during online ms submission.

- The FUNDING INFO needs to be part of the Acknowledgments, so no separate Funding title heading is needed.

- Table EV1 is a dataset and should be updated to Dataset EV1 in all places (in the file and ms callouts).

- The Materials and Methods section should include a separate Reagents and Tools Table file (listing key reagents, experimental models, software and relevant equipment and including their sources and relevant identifiers) and a Methods and Protocols section in which methods should be described using a step-by-step protocol format with bullet points. More information and a downloadable Table template is available in our guide to authors online: https://link.springer.com/journal/44319/submission-guidelines

- The Source Data files need to be re-organized into folders: one folder per main figure with the panels of this figure in this folder needs to be uploaded.

- The manuscript sections should be in the following order: Title page - Abstract & Keywords - Introduction - Results - Discussion - Methods - Data Availability - Acknowledgments - Disclosure Statement & Competing Interests - References - Figure Legends - (Main Tables with legends if applicable) - Expanded View Figure Legends.

- Materials and methods should be renamed to Methods

- The correct nomenclature of EV figures is only present in the titles of the figures in our online system. It needs to be updated to Figure EV1, etc. also in the source file names, individual figure files, legends in the ms and ms callouts.

* Figure Legends - Comments *

- Please note that the exact p values are not provided in the legends of figures 3I, J; 5C, D; 6C, please provide exact values as reasonable.
- Please indicate the statistical test used for data analysis in the legends of figures 6A, B, D
- Please note that the box plots need to be defined in terms of minima, maxima, centre, bounds of box and whiskers, and percentile in the legend of figure 1D
- Please note that information related to n is missing in the legend of figure 4D
- Please note that the scale bar is missing for figures 3A', B', C', D'
- Please note that scale bar and its definition are missing for figures 3E-G.

Please correct this sentence in the abstract to present tense: Here, we investigated .... to Here, we investigate...

EMBO press papers are accompanied online by A) a short (1-2 sentences) summary of the findings and their significance, B) 2-3 bullet points highlighting key results and C) a synopsis image that is exactly 550 pixels wide and 300 pixels high. The synopsis image should provide a sketch of the major findings, like a graphical abstract. Please note that text needs to be readable at the final size. Please send us this information along with the final manuscript.

I look forward to seeing a final version of your manuscript as soon as possible.

Best regards,
Esther

Esther Schnapp, PhD
Senior Editor
EMBO reports

Referee #1:

The authors performed all the experiments that I requested in my first review. While they unfortunately did not provide any additional insights, I believe it may still help them in future work. Also, as requested a proper control was now included for one of the experiments.

Dear Editor and the editorial office,

Thank you very much for the positive evaluation of our manuscript and for the helpful editorial comments. We sincerely apologize for the considerable delay in submitting our revised manuscript.

We have revised the manuscript and associated files according to all requests. In addition, as previously discussed with and approved by the editor, we corrected an error identified in the RNA-seq analysis and revised the manuscript accordingly. The revised manuscript was shared with the editor prior to this re-submission, and we received confirmation that these changes could be incorporated into the final revision.

All modifications, including those related to the RNA-seq analysis, are highlighted in blue in the revised manuscript. Our point-by-point responses are provided below. Comments from the Editorial Office are shown in bold sans-serif font, and our responses are shown in regular serif font.

1. Conflict of interest subheading

Comment: Please correct the conflict of interest subheading to "Disclosure and Competing Interests Statement".

Response: The subheading has been corrected to "Disclosure and Competing Interests Statement".

----

2. Author Credits

Comment: The author credits need to be removed from the ms file. All credits need to be entered during online ms submission.

Response: The author credits have been removed from the manuscript and entered through the online submission system.

----

3. Funding Information

Comment: The FUNDING INFO needs to be part of the Acknowledgments, so no separate Funding title heading is needed.

Response: The funding information has been moved to the Acknowledgments section, and the separate Funding heading has been removed.

----

4. Table EV1

Comment: Table EV1 is a dataset and should be updated to Dataset EV1 in all places (in the file and ms callouts).

Response: All references to Table EV1 have been revised to Dataset EV1 throughout the manuscript and associated files.

----

5. Reagents and Tools Table and Methods and Protocols section

Comment: The Materials and Methods section should include a separate Reagents and Tools Table file (listing key reagents, experimental models, software and relevant equipment and including their sources and relevant identifiers) and a Methods and Protocols section in which methods should be described using a step-by-step protocol format with bullet points.

Response: A separate Reagents and Tools Table has been prepared and incorporated into the Methods section. In addition, a Methods and Protocols section describing the experimental procedures in a step-by-step format has been added.
Regarding the recommendation to use bullet points, we contacted the editorial office for clarification and were informed that the use of bullet points is not mandatory. We therefore followed the format used in previously published EMBO Reports articles.

----

6. Source Data organization

Comment: The Source Data files need to be re-organized into folders: one folder per main figure with the panels of this figure in this folder needs to be uploaded.

Response: The Source Data files have been reorganized into separate folders, with one folder for each main figure containing all corresponding panels.

----

7. Manuscript section order

Comment: The manuscript sections should be in the following order: Title page - Abstract & Keywords - Introduction - Results - Discussion - Methods - Data Availability - Acknowledgments - Disclosure Statement & Competing Interests - References - Figure Legends - (Main Tables with legends if applicable) - Expanded View Figure Legends.

Response: The manuscript has been reorganized according to the requested section order.

----

8. Methods section

Comment: Materials and methods should be renamed to Methods

Response: The section title has been changed from Materials and Methods to Methods.

----

9. Expanded View figure nomenclature

Comment: The correct nomenclature of EV figures is only present in the titles of the figures in our online system. It needs to be updated to Figure EV1, etc. also in the source file names, individual figure files, legends in the ms and ms callouts.

Response: The nomenclature has been revised consistently throughout the manuscript, figure legends, figure files, source file names, and callouts.

----

10. Figure Legends

- Please note that the exact p values are not provided in the legends of figures 3I, J; 5C, D; 6C, please provide exact values as reasonable.

Response: We consulted the editor regarding this point and were informed that exact p values are not required when $p < 0.0001$. We have therefore followed this guidance.

- Please indicate the statistical test used for data analysis in the legends of figures 6A, B, D.

Response: The statistical tests used for data analysis have been added to the corresponding figure legends.

- Please note that the box plots need to be defined in terms of minima, maxima, centre, bounds of box and whiskers, and percentile in the legend of figure 1D.

Response: The figure legend has been revised to define the box plots. We also added a Source Data file containing all numerical values.

- Please note that information related to n is missing in the legend of figure 4D.

Response: The sample size information has been added to the figure legend.

- Please note that the scale bar is missing for figures 3A', B', C', D'.

Response: Scale bars have been added to these figures.

- Please note that scale bar and its definition are missing for figures 3E-G.

Response: Scale bars and their corresponding definitions have been added.

----

11. Others

Comment: Please correct this sentence in the abstract to present tense: Here, we investigated .... to Here, we investigate...

Response: The sentence has been revised to "Here, we investigate…".

Comment: EMBO press papers are accompanied online by A) a short (1-2 sentences) summary of the findings and their significance, B) 2-3 bullet points highlighting key results and C) a synopsis image that is exactly 550 pixels wide and 300 pixels high. The synopsis image should provide a sketch of the major findings, like a graphical abstract. Please note that text needs to be readable at the final size. Please send us this information along with the final manuscript.

Response: A short summary, key results, and a synopsis image have been prepared and submitted along with the revised manuscript.

Additional revisions related to the RNA-seq analysis are described below. As noted above, the details below were previously communicated to the editor and approved. For reference, the following message was sent to the editor on May 24, and we received a response on June 8.

While revising the Methods section according to your instructions, we realized that, in our RNA-seq analysis, normalized FPKM values had inadvertently been used instead of raw count data. We therefore repeated the analysis using the correct raw count data and revised the relevant figures accordingly, resulting in corresponding changes to Figures 1 and 2, as well as Figures EV2 and EV3.

The major revisions are summarized as follows:

(1) Correction of the clustering analysis in Figure 1D
Re-analysis using the correct raw count data resulted in partial changes to the gene grouping shown in Figure 1D. During this process, we found that the Day 3 samples did not show consistent differences between the two conditions, and their inclusion reduced our ability to detect biologically important differential gene expression at Day 6. This result also suggests that a 3-day feeding period may be insufficient to produce robust transcriptional differences between the two conditions. We therefore performed the clustering analysis after excluding the Day 3 samples, and this has been clearly stated in the Methods section. Importantly, the key finding that cell adhesion–related genes are upregulated under unfed conditions remains unchanged.

(2) Re-analysis of downstream KEGG and GO analyses (Figures 1E, 1F, and Figure 2)
Because the KEGG and GO analyses were originally performed based on the grouping shown in Figure 1D, we repeated these analyses using the corrected gene grouping. As a result, Figures 1E, 1F, and Figure 2 have been updated accordingly.

(3) Revision of related Expanded View figures
The Expanded View figures associated with Figures 1 and 2 have also been updated accordingly. In addition, because Figure EV3 became overly complex, the graphs showing expression changes of cell adhesion–related genes based on FPKM values, together with the table of thyroid-specific genes, have been separated and are now presented as Figure EV4.

We thank the editor and referee for their careful evaluation of our work and helpful comments.

Sincerely,
Asako Shindo

Dr. Asako Shindo
The University of Osaka
Biological Sciences
Machikaneyama-cho 1-1
Toyonaka, Osaka 560-0043
Japan

Dear Dr. Shindo,

I am very pleased to accept your manuscript for publication in the next available issue of EMBO reports. Thank you for your contribution to our journal.

Your manuscript will be processed for publication by EMBO Press. It will be copy edited and you will receive page proofs prior to publication. Please note that you will be contacted by Springer Nature Author Services to complete licensing and payment information.

You may qualify for financial assistance for your publication charges - either via a Springer Nature fully open access agreement or an EMBO initiative. Check your eligibility: https://link.springer.com/journal/44319/how-to-publish-with-us

Should you be planning a Press Release on your article, please notify the production team via this form in order to coordinate publication and release dates: https://forms.cloud.microsoft/e/eH4A8JVP6g

If you have any questions, please do not hesitate to contact the Editorial Office. Thank you for your contribution to EMBO Reports.

Best regards,
Esther

Esther Schnapp, PhD
Senior Editor
EMBO reports

>>> Please note that it is EMBO Reports policy for the transcript of the editorial process (containing referee reports and your response letter) to be published as an online supplement to each paper. If you do NOT want this, you will need to inform the Editorial Office via email immediately. More information is available here: https://link.springer.com/partners/embo-press/editorial-policies#Peer%20review

EMBO Press Author Checklist

Corresponding Author Name: Asako Shindo	
Journal Submitted to: EMBO Reports	
Manuscript Number: EMBOR-2025-62377-T	

USEFUL LINKS FOR COMPLETING THIS FORM
The EMBO Journal - Author Guidelines
EMBO Reports - Author Guidelines
Molecular Systems Biology - Author Guidelines
EMBO Molecular Medicine - Author Guidelines

Reporting Checklist for Life Science Articles (updated January
This checklist is adapted from Materials Design Analysis Reporting (MDAR) Checklist for Authors. MDAR establishes a minimum set of requirements in transparent reporting in the life sciences (see Statement of Task: 10.31222/osf.io/9sm4x). Please follow the journal's guidelines in preparing your
Please note that a copy of this checklist will be published alongside your article.

Abridged guidelines for figures
1. Data
The data shown in figures should satisfy the following conditions:
- the data were obtained and processed according to the field's best practice and are presented to reflect the results of the experiments in an accurate and unbiased manner.
- ideally, figure panels should include only measurements that are directly comparable to each other and obtained with the same assay.
- plots include clearly labeled error bars for independent experiments and sample sizes. Unless justified, error bars should not be shown for technical
- if n<5, the individual data points from each experiment should be plotted. Any statistical test employed should be justified.
- Source Data should be included to report the data underlying figures according to the guidelines set out in the authorship guidelines on Data

2. Captions
Each figure caption should contain the following information, for each panel where they are relevant:
- a specification of the experimental system investigated (eg cell line, species name).
- the assay(s) and method(s) used to carry out the reported observations and measurements.
- an explicit mention of the biological and chemical entity(ies) that are being measured.
- an explicit mention of the biological and chemical entity(ies) that are altered/varied/perturbed in a controlled manner.
- the exact sample size (n) for each experimental group/condition, given as a number, not a range;
- a description of the sample collection allowing the reader to understand whether the samples represent technical or biological replicates (including how many animals, litters, cultures, etc.).
- a statement of how many times the experiment shown was independently replicated in the laboratory.
- definitions of statistical methods and measures:
  - common tests, such as t-test (please specify whether paired vs. unpaired), simple χ2 tests, Wilcoxon and Mann-Whitney tests, can be unambiguously identified by name only, but more complex techniques should be described in the methods section;
  - are tests one-sided or two-sided?
  - are there adjustments for multiple comparisons?
  - exact statistical test results, e.g., P values = x but not P values < x;
  - definition of 'center values' as median or average;
  - definition of error bars as s.d. or s.e.m.

Please complete ALL of the questions below.
Select "Not Applicable" only when the requested information is not relevant for your study.

Materials

Newly Created Materials	Information included in the manuscript?	In which section is the information available? (Reagents and Tools Table, Materials and Methods, Figures, Data Availability Section)
New materials and reagents need to be available; do any restrictions apply?	Not Applicable	

Antibodies	Information included in the manuscript?	In which section is the information available? (Reagents and Tools Table, Materials and Methods, Figures, Data Availability Section)
For **antibodies** provide the following information:<br>- Commercial antibodies: RRID (if possible) or supplier name, catalogue number and or/clone number<br>- Non-commercial: RRID or citation	Yes	GA509; Dako, Berlin, Germany does not have RRID.

DNA and RNA sequences	Information included in the manuscript?	In which section is the information available? (Reagents and Tools Table, Materials and Methods, Figures, Data Availability Section)
**Short novel DNA or RNA including primers, probes**: provide the sequences.	Not Applicable	

Cell materials	Information included in the manuscript?	In which section is the information available? (Reagents and Tools Table, Materials and Methods, Figures, Data Availability Section)
**Cell lines:** Provide species information, strain. Provide accession number in repository **OR** supplier name, catalog number, clone number, and/**OR** RRID.	Not Applicable	
**Primary cultures:** Provide species, strain, sex of origin, genetic modification status.	Not Applicable	
Report if the cell lines were recently **authenticated** (e.g., by STR profiling) and tested for mycoplasma contamination.	Not Applicable	

Experimental animals	Information included in the manuscript?	In which section is the information available? (Reagents and Tools Table, Materials and Methods, Figures, Data Availability Section)
**Laboratory animals or Model organisms:** Provide species, strain, sex, age, genetic modification status. Provide accession number in repository **OR** supplier name, catalog number, clone number, **OR** RRID.	Yes	
**Animal observed in or captured from the field:** Provide species, sex, and age where possible.	Yes	
Please detail **housing and husbandry conditions**.	Yes	

Plants and microbes	Information included in the manuscript?	In which section is the information available? (Reagents and Tools Table, Materials and Methods, Figures, Data Availability Section)
**Plants:** provide species and strain, ecotype and cultivar where relevant, unique accession number if available, and source (including location for collected wild specimens).	Not Applicable	
**Microbes:** provide species and strain, unique accession number if available, and source.	Not Applicable	

Human research participants	Information included in the manuscript?	In which section is the information available? (Reagents and Tools Table, Materials and Methods, Figures, Data Availability Section)
If collected and within the bounds of privacy constraints report on age, sex and gender or ethnicity for all study participants.	Not Applicable	

Core facilities	Information included in the manuscript?	In which section is the information available? (Reagents and Tools Table, Materials and Methods, Figures, Data Availability Section)
If your work benefited from core facilities, was their service mentioned in the acknowledgments section?	Not Applicable	

Design

**Study protocol**	**Information included in the manuscript?**	**In which section is the information available?** (Reagents and Tools Table, Materials and Methods, Figures, Data Availability Section)
If study protocol has been **pre-registered, provide DOI in the manuscript**. For clinical trials, provide the trial registration number **OR** cite DOI.	Not Applicable	
Report the **clinical trial registration number** (at ClinicalTrials.gov or equivalent), where applicable.	Not Applicable	

**Laboratory protocol**	**Information included in the manuscript?**	**In which section is the information available?** (Reagents and Tools Table, Materials and Methods, Figures, Data Availability Section)
Provide DOI OR other citation details if **external detailed step-by-step protocols** are available.	Not Applicable	

**Experimental study design and statistics**	**Information included in the manuscript?**	**In which section is the information available?** (Reagents and Tools Table, Materials and Methods, Figures, Data Availability Section)
Include a statement about **sample size** estimate even if no statistical methods were used.	Yes	
Were any steps taken to minimize the effects of subjective bias when allocating animals/samples to treatment (e.g. **randomization procedure**)? If yes, have they been described?	Yes	
Include a statement about **blinding** even if no blinding was done.	Yes	
Describe **inclusion/exclusion criteria** if samples or animals were excluded from the analysis. Were the criteria pre-established?<br><br>If sample or data points were omitted from analysis, report if this was due to attrition or intentional exclusion and provide justification.	Not Applicable	
For every figure, are **statistical tests** justified as appropriate? Do the data meet the assumptions of the tests (e.g., normal distribution)? Describe any methods used to assess it. Is there an estimate of variation within each group of data? Is the variance similar between the groups that are being statistically compared?	Yes	

**Sample definition and in-laboratory replication**	**Information included in the manuscript?**	**In which section is the information available?** (Reagents and Tools Table, Materials and Methods, Figures, Data Availability Section)
In the figure legends: state number of times the experiment was **replicated** in laboratory.	Yes	
In the figure legends: define whether data describe **technical or biological replicates**.	Yes	

Ethics

**Ethics**	**Information included in the manuscript?**	**In which section is the information available?** (Reagents and Tools Table, Materials and Methods, Figures, Data Availability Section)
Studies involving **human participants**: State details of **authority granting ethics approval** (IRB or equivalent committee(s), provide reference number for approval.	Not Applicable	
Studies involving **human participants**: Include a statement confirming that **informed consent** was obtained from all subjects and that the experiments conformed to the principles set out in the WMA Declaration of Helsinki and the Department of Health and Human Services Belmont Report.	Not Applicable	
Studies involving **human participants:** For publication of **patient photos**, include a statement confirming that consent to publish was obtained.	Not Applicable	
Studies involving experimental **animals**: State details of **authority granting ethics approval** (IRB or equivalent committee(s), provide reference number for approval. Include a statement of compliance with ethical regulations.	Yes	
Studies involving **specimen and field samples:** State if relevant **permits** obtained, provide details of authority approving study; if none were required, explain why.	Not Applicable	

**Dual Use Research of Concern (DURC)**	**Information included in the manuscript?**	**In which section is the information available?** (Reagents and Tools Table, Materials and Methods, Figures, Data Availability Section)
Could your study fall under dual use research restrictions? Please check biosecurity documents and list of **select agents and toxins** (CDC): https://www.selectagents.gov/sat/list.htm	Not Applicable	
If you used a select agent, is the security level of the lab appropriate and reported in the manuscript?	Not Applicable	
If a study is subject to dual use research of concern regulations, is the name of the **authority granting approval and reference number** for the regulatory approval provided in the manuscript?	Not Applicable	

Reporting

The MDAR framework recommends adoption of discipline-specific guidelines, established and endorsed through community initiatives. Journals have their own policy about requiring specific guidelines and recommendations to complement MDAR.

**Adherence to community standards**	**Information included in the manuscript?**	**In which section is the information available?** (Reagents and Tools Table, Materials and Methods, Figures, Data Availability Section)
State if relevant guidelines or checklists (e.g., **ICMJE, MIBBI, ARRIVE, PRISMA**) have been followed or provided.	Not Applicable	
For **tumor marker prognostic studies,** we recommend that you follow the **REMARK** reporting guidelines (see link list at top right). See author guidelines, under 'Reporting Guidelines'. Please confirm you have followed these guidelines.	Not Applicable	
For **phase II and III randomized controlled trials**, please refer to the **CONSORT** flow diagram (see link list at top right) and submit the CONSORT checklist (see link list at top right) with your submission. See author guidelines, under 'Reporting Guidelines'. Please confirm you have submitted this list.	Not Applicable	

Data Availability

**Data availability**	**Information included in the manuscript?**	**In which section is the information available?** (Reagents and Tools Table, Materials and Methods, Figures, Data Availability Section)
Have **primary datasets** been deposited according to the journal's guidelines (see 'Data Deposition' section) and the respective accession numbers provided in the Data Availability Section?	Yes	
Were **human clinical and genomic datasets** deposited in a public access-controlled repository in accordance to ethical obligations to the patients and to the applicable consent agreement?	Not Applicable	
Are **computational models** that are central and integral to a study available without restrictions in a machine-readable form? Were the relevant accession numbers or links provided?	Not Applicable	
If publicly available data were reused, provide the respective **data citations in the reference list**.	Not Applicable	
