# Supplementary material for: Glycolytic suppression of cell adhesion promotes thyroid morphogenesis in response to nutrition
Source: EMBO Rep. 2026 Jul 20;27(17):5143–61. doi: 10.1038/s44319-026-00868-4 (PMC13554297; doi:10.1038/s44319-026-00868-4)
Supplement: Supplementary file 2 [file 44319_2026_868_MOESM8_ESM.pdf]

Expanded View Figures

A

			Total Reads	Total Alignments	Mapped Alignments	Mapped Alignments [%]	MQ0 [%]	RNA concentration (ng/µl)	RIN
Sample #1	Unfed	Day0	83556180	89955256	72245124	80.31	13.66	2.9	7.9
		Day3	96204108	101361642	79732430	78.66	10.14	3.9	7.7
		Day6	93320344	97587958	75921236	77.8	8.942	1.6	7.6
		Day9	75673376	79807618	60453196	75.75	10.55	2.0	7.2
	Fed	Day3	89452422	95241534	73185048	76.84	12.26	10.6	7.0
		Day6	87193038	91872292	72596384	79.02	10.49	6.9	7.3
		Day9	77908586	81897038	59854098	73.08	10.87	1.3	6.7
Sample #2	Unfed	Day0	82730472	88379188	67214848	76.05	12.77	6.7	6.9
		Day3	80240102	84747680	65312616	77.07	10.69	3.1	6.9
		Day6	80752754	84676984	67808626	80.08	9.042	2.9	7.3
	Fed	Day3	88826314	94667278	71731110	75.77	12.43	2.1	6.5
		Day6	81082128	85686058	66522568	77.64	11.04	4.4	7.2

B

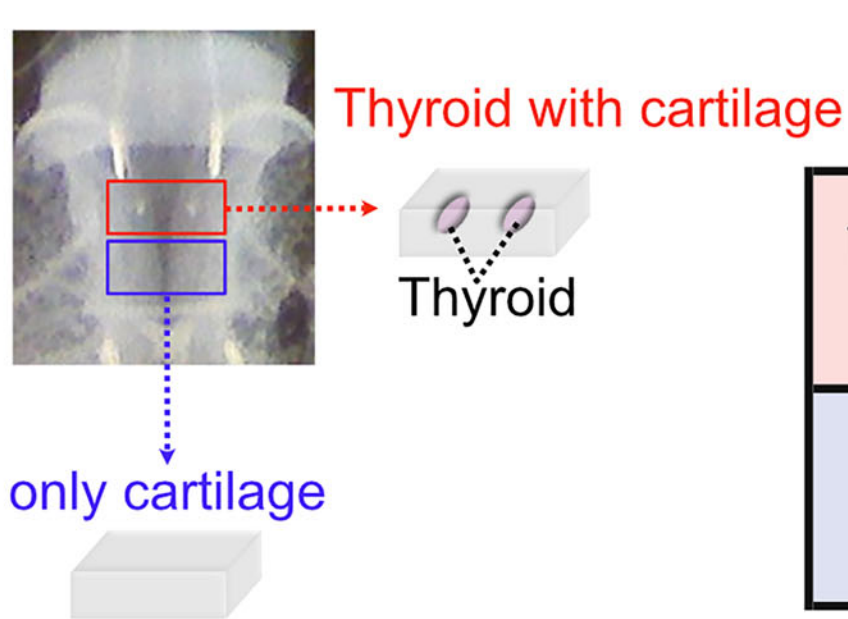

		Total Reads	Mapped Alignments	Mapped Alignments [%]	MQ0 [%]
Thyroid with Cartilage	Day0	12,066,973	10,614,720	87.97%	10.06%
	Day3	9,907,928	8,578,922	86.59%	11.05%
	Day6	11,422,249	9,683,106	84.77	12.92%
Cartilage	Day0	13,829,160	11,598,392	83.87%	13.92%
	Day3	12,867,752	10,408,129	80.89%	16.51%
	Day6	11,804,441	9,492,977	80.42%	17.08%

C

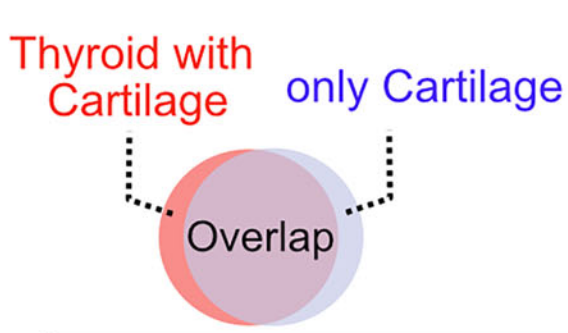

	Day 0		Day 3		Day 6	
	Number	Percentage	Number	Percentage	Number	Percentage
Thyroid with cartilage	1202	2.5%	1768	3.7%	1467	3.2%
Overlap	45632	94.2%	44806	93.9%	43828	94.3%
only Cartilage	1629	3.4%	1155	2.4%	1160	2.5%

Figure EV1. Related to Fig. 1.

(**A**) Summary of RNA-seq quality metrics generated in this study. The listed parameters include total reads, total alignments, mapped alignments, the percentage of mapped alignments, the percentage of reads with mapping quality 0 (MQ0), RNA concentration, and RNA integrity number (RIN). (**B**) The thyroid or cartilage region used for the RNA-seq analysis. The table shows RNA-seq quality metrics used for comparison between two different regions, thyroid with cartilage and cartilage. (**C**) The table displaying the overlap of expressed genes in the thyroid region (red) and cartilage region (blue) at Days 0, 3, and 6. RNA was extracted from unfed larvae, and genes with raw counts ≤5 were excluded from the analysis. Percentages represent the proportion of genes in each category relative to the total number detected at each time point.

© The Author(s)

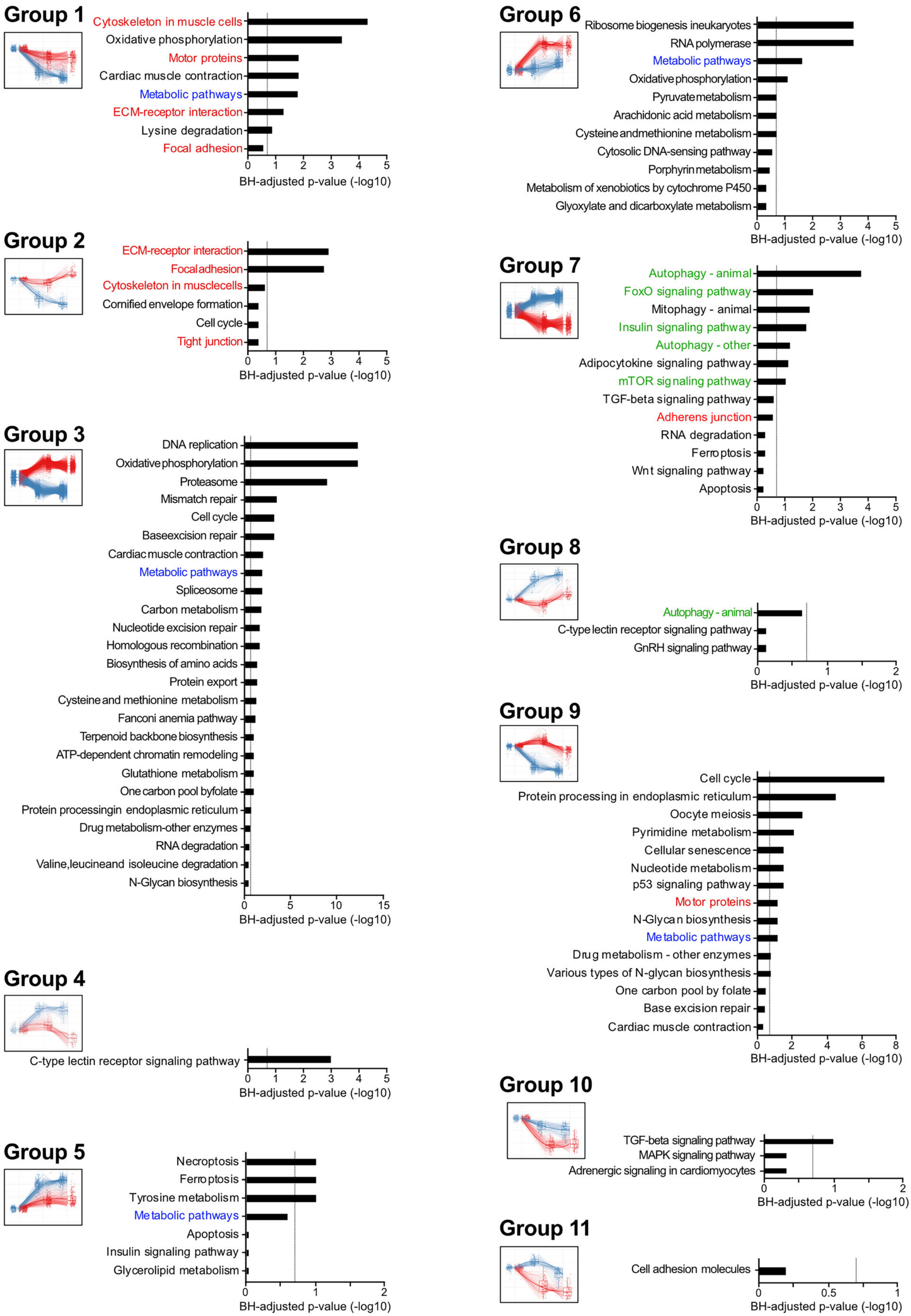

Group 1
Cytoskeleton in muscle cells
Oxidative phosphorylation
Motor proteins
Cardiac muscle contraction
Metabolic pathways
ECM-receptor interaction
Lysine degradation
Focal adhesion
BH-adjusted p-value (-log10)
Group 2
ECM-receptor interaction
Focal adhesion
Cytoskeleton in muscle cells
Cornified envelope formation
Cell cycle
Tight junction
BH-adjusted p-value (-log10)
Group 3
DNA replication
Oxidative phosphorylation
Proteasome
Mismatch repair
Cell cycle
Base excision repair
Cardiac muscle contraction
Metabolic pathways
Spliceosome
Carbon metabolism
Nucleotide excision repair
Homologous recombination
Biosynthesis of amino acids
Protein export
Cysteine and methionine metabolism
Fanconi anemia pathway
Terpenoid backbone biosynthesis
ATP-dependent chromatin remodeling
Glutathione metabolism
One carbon pool by folate
Protein processing in endoplasmic reticulum
Drug metabolism-other enzymes
RNA degradation
Valine, leucine and isoleucine degradation
N-Glycan biosynthesis
BH-adjusted p-value (-log10)
Group 4
C-type lectin receptor signaling pathway
BH-adjusted p-value (-log10)
Group 5
Necroptosis
Ferroptosis
Tyrosine metabolism
Metabolic pathways
Apoptosis
Insulin signaling pathway
Glycerolipid metabolism
BH-adjusted p-value (-log10)
Group 6
Ribosome biogenesis in eukaryotes
RNA polymerase
Metabolic pathways
Oxidative phosphorylation
Pyruvate metabolism
Arachidonic acid metabolism
Cysteine and methionine metabolism
Cytosolic DNA-sensing pathway
Porphyrin metabolism
Metabolism of xenobiotics by cytochrome P450
Glyoxylate and dicarboxylate metabolism
BH-adjusted p-value (-log10)
Group 7
Autophagy - animal
FoxO signaling pathway
Mitophagy - animal
Insulin signaling pathway
Autophagy - other
Adipocytokine signaling pathway
mTOR signaling pathway
TGF-beta signaling pathway
Adherens junction
RNA degradation
Ferroptosis
Wnt signaling pathway
Apoptosis
BH-adjusted p-value (-log10)
Group 8
Autophagy - animal
C-type lectin receptor signaling pathway
GnRH signaling pathway
BH-adjusted p-value (-log10)
Group 9
Cell cycle
Protein processing in endoplasmic reticulum
Oocyte meiosis
Pyrimidine metabolism
Cellular senescence
Nucleotide metabolism
p53 signaling pathway
Motor proteins
N-Glycan biosynthesis
Metabolic pathways
Drug metabolism - other enzymes
Various types of N-glycan biosynthesis
One carbon pool by folate
Base excision repair
Cardiac muscle contraction
BH-adjusted p-value (-log10)
Group 10
TGF-beta signaling pathway
MAPK signaling pathway
Adrenergic signaling in cardiomyocytes
BH-adjusted p-value (-log10)
Group 11
Cell adhesion molecules
BH-adjusted p-value (-log10)

Figure EV2. Related to Figs. 1 and 2.

KEGG pathway analysis for each group in Fig. 1D. Bar plots show enriched pathways (*x* axis: −log10(BH-adjusted *P* values) for representative groups. Selected pathways are color-coded to highlight categories related to cell behaviors (red), metabolism (blue), and starvation-related genes (green).

Group 1

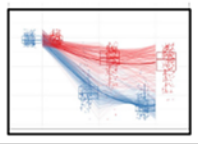

GO ID	Term	Count	–log10(BH)	Genes (%)
GO:0015990	electron transport coupled proton transport	3	1.401	2.17
GO:0009060	aerobic respiration	3	0.821	2.17
GO:0030199	collagen fibril organization	3	0.453	2.17
GO:0006120	mitochondrial electron transport, NADH to ubiquinone	3	0.453	2.17
GO:0006936	muscle contraction	3	0.441	2.17
GO:0032981	mitochondrial respiratory chain complex I assembly	3	0.441	2.17
GO:0061975	articular cartilage development	2	0.175	1.45
GO:0006457	protein folding	4	0.134	2.9
GO:0042773	ATP synthesis coupled electron transport	2	0.134	1.45
GO:0007015	actin filament organization	4	0.106	2.9
GO:0034501	protein localization to kinetochore	2	0.106	1.45
GO:0009617	response to bacterium	2	0.106	1.45
GO:0060348	bone development	2	0.106	1.45
GO:0007076	mitotic chromosome condensation	2	0.102	1.45
GO:0007399	nervous system development	6	0.000	4.35
GO:0007417	central nervous system development	3	0.000	2.17
GO:0001558	regulation of cell growth	2	0.000	1.45
GO:0007528	neuromuscular junction development	2	0.000	1.45

Group 2

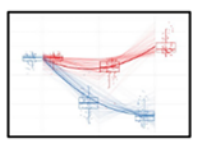

GO ID	GO termes	Count	–log10(BH)	%
GO:0030198	extracellular matrix organization	4	1.358	12.19
GO:0051754	meiotic sister chromatid cohesion, centromeric	2	0.616	6.45
GO:0008652	amino acid biosynthetic process	2	0.210	6.45

Group 3

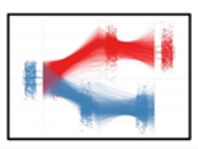

GO ID	GO term	Count	–log10(BH)	%
GO:0006260	DNA replication	29	15.590	4.63
GO:0000398	mRNA splicing, via spliceosome	25	9.250	3.99
GO:0006270	DNA replication initiation	12	8.017	1.92
GO:0008380	RNA splicing	27	5.967	4.31
GO:0000727	double-strand break repair via break-induced replication	9	5.959	1.44
GO:0006457	protein folding	18	5.124	2.88
GO:0042744	hydrogen peroxide catabolic process	11	4.971	1.76
GO:0006261	DNA-templated DNA replication	8	4.635	1.28
GO:0044786	cell cycle DNA replication	6	4.635	0.96
GO:0051603	proteolysis involved in protein catabolic process	13	4.481	2.08
GO:0015671	oxygen transport	9	4.457	1.44
GO:0006397	mRNA processing	27	3.914	4.31
GO:0000387	spliceosomal snRNP assembly	8	3.582	1.28
GO:0006123	mitochondrial electron transport, cytochrome c to oxygen	8	3.426	1.28
GO:0006281	DNA repair	23	3.345	3.67
GO:0048821	erythrocyte development	7	3.150	1.12
GO:0030174	regulation of DNA-templated DNA replication initiation	7	3.150	1.12
GO:0006119	oxidative phosphorylation	6	2.876	0.96
GO:0043161	proteasome-mediated ubiquitin-dependent protein catabolic process	19	2.873	3.04
GO:0051301	cell division	26	2.730	4.15
GO:0006412	translation	23	2.721	3.67
GO:0006271	DNA strand elongation involved in DNA replication	5	2.664	0.8
GO:0006338	chromatin remodeling	13	2.221	2.08
GO:0006279	premeiotic DNA replication	4	2.221	0.64
GO:0006413	translational initiation	10	2.175	1.6
GO:0051664	nuclear pore localization	4	1.839	0.64
GO:0090435	protein localization to nuclear envelope	4	1.839	0.64
GO:0006974	DNA damage response	20	1.804	3.19
GO:0030150	protein import into mitochondrial matrix	6	1.775	0.96
GO:0006298	mismatch repair	5	1.775	0.8
GO:0006122	mitochondrial electron transport, ubiquinol to cytochrome c	5	1.712	0.8
GO:0051028	mRNA transport	9	1.688	1.44
GO:0006511	ubiquitin-dependent protein catabolic process	15	1.532	2.4
GO:0015031	protein transport	30	1.352	4.79
GO:0008202	steroid metabolic process	9	1.331	1.44
GO:0045041	protein import into mitochondrial intermembrane space	3	1.246	0.48
GO:0007017	microtubule-based process	7	1.243	1.12
GO:0045039	protein insertion into mitochondrial inner membrane	4	1.217	0.64
GO:0006284	base-excision repair	5	1.145	0.8
GO:0006998	nuclear envelope organization	4	1.145	0.64
GO:0031507	heterochromatin formation	8	1.108	1.28
GO:0006730	one-carbon metabolic process	5	1.108	0.8
GO:0006614	SRP-dependent cotranslational protein targeting to membrane	4	1.020	0.64
GO:0046653	tetrahydrofolate metabolic process	3	0.807	0.48
GO:0006406	mRNA export from nucleus	5	0.785	0.8
GO:0045292	mRNA cis splicing, via spliceosome	4	0.728	0.64
GO:0042254	ribosome biogenesis	8	0.712	1.28

Group 4

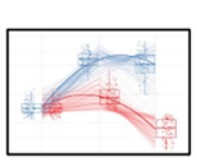

GO ID	GO term	Count	–log10(BH)	Genes (%)
GO:0006357	regulation of transcription by RNA polymerase II	12	0.391	18.46
GO:0006355	regulation of DNA-templated transcription	9	0.391	13.85
GO:0045944	positive regulation of transcription by RNA polymerase II	5	0.391	7.69
GO:0003100	regulation of systemic arterial blood pressure by endothelin	2	0.391	3.08
GO:0014826	vein smooth muscle contraction	2	0.313	3.08
GO:0045987	positive regulation of smooth muscle contraction	2	0.235	3.08
GO:0046931	pore complex assembly	2	0.163	3.08
GO:0019229	regulation of vasoconstriction	2	0.133	3.08
GO:0042310	vasoconstriction	2	0.133	3.08
GO:0097746	blood vessel diameter maintenance	2	0.093	3.08
GO:0030148	sphingolipid biosynthetic process	2	0.093	3.08
GO:0001501	skeletal system development	2	0.093	3.08

Group 5

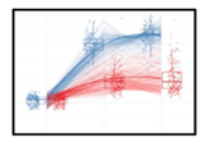

GO ID	GO term	Count	–log10(BH)	%
GO:0006572	L-tyrosine catabolic process	3	0.873	2.52
GO:0006559	L-phenylalanine catabolic process	3	0.873	2.52
GO:0001867	complement activation, lectin pathway	3	0.614	2.52
GO:0006826	iron ion transport	3	0.431	2.52
GO:0042632	cholesterol homeostasis	3	0.101	2.52
GO:0006508	proteolysis	8	0	6.72
GO:0006955	immune response	5	0	4.2
GO:0006915	apoptotic process	5	0	4.2
GO:0005975	carbohydrate metabolic process	4	0	3.36
GO:0009968	negative regulation of signal transduction	3	0	2.52
GO:0016042	lipid catabolic process	3	0	2.52
GO:0034067	protein localization to Golgi apparatus	2	0	1.68
GO:0006000	fructose metabolic process	2	0	1.68
GO:0042438	melanin biosynthetic process	2	0	1.68
GO:0019731	antibacterial humoral response	2	0	1.68
GO:0006956	complement activation	2	0	1.68
GO:0034375	high-density lipoprotein particle remodeling	2	0	1.68
GO:0043122	regulation of canonical NF-kappaB signal transduction	2	0	1.68

Group 6

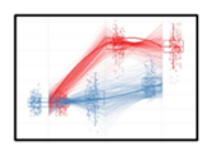

GO ID	GO term	Count	–log10(BH)	%
GO:0006364	rRNA processing	18	14.914	10.65
GO:0042254	ribosome biogenesis	14	11.127	8.28
GO:0032259	methylation	9	2.298	5.33
GO:0006396	RNA processing	7	2.955	4.14
GO:0042273	ribosomal large subunit biogenesis	6	3.380	3.55
GO:0006351	DNA-templated transcription	6	1.924	3.55
GO:0006457	protein folding	6	1.282	3.55
GO:0008380	RNA splicing	6	0.339	3.55
GO:0042274	ribosomal small subunit biogenesis	5	2.483	2.96
GO:0000463	maturation of LSU-rRNA from tricistronic rRNA transcript (SSU-rRNA, 5.8S rRNA, LSU-rRNA)	4	1.790	2.37
GO:0007005	mitochondrion organization	4	0.924	2.37
GO:0000462	maturation of SSU-rRNA from tricistronic rRNA transcript (SSU-rRNA, 5.8S rRNA, LSU-rRNA)	3	0.633	1.78
GO:0019752	carboxylic acid metabolic process	3	0.633	1.78
GO:0051135	positive regulation of NK T cell activation	2	0.633	1.18
GO:0031118	rRNA pseudouridine synthesis	2	0.339	1.18
GO:0000479	endonucleolytic cleavage of tricistronic rRNA transcript (SSU-rRNA, 5.8S rRNA, LSU-rRNA)	2	0.339	1.18
GO:0045345	positive regulation of MHC class I biosynthetic process	2	0.339	1.18
GO:0031120	snRNA pseudouridine synthesis	2	0.302	1.18
GO:0042256	cytosolic ribosome assembly	2	0.203	1.18
GO:0000447	endonucleolytic cleavage in ITS1 to separate SSU-rRNA from 5.8S rRNA and LSU-rRNA from tricistronic rRNA transcript (SSU-rRNA, 5.8S rRNA, LSU-rRNA)	2	0.196	1.18
GO:0000466	maturation of 5.8S rRNA from tricistronic rRNA transcript (SSU-rRNA, 5.8S rRNA, LSU-rRNA)	2	0.196	1.18
GO:0019372	lipoxygenase pathway	2	0.191	1.18
GO:0006108	malate metabolic process	2	0.191	1.18
GO:0034440	lipid oxidation	2	0.169	1.18
GO:0031167	rRNA methylation	2	0.150	1.18
GO:0001510	RNA methylation	2	0.150	1.18
GO:0072344	rescue of stalled ribosome	2	0.150	1.18
GO:0006360	transcription by RNA polymerase I	2	0.135	1.18
GO:0000460	maturation of 5.8S rRNA	2	0.084	1.18
GO:0000470	maturation of LSU-rRNA	2	0.084	1.18

Group 7

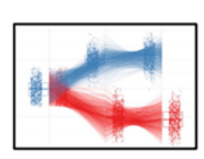

GO ID	GO term	Count	–log10(BH)	%
GO:0045892	negative regulation of DNA-templated transcription	14	1.996	3.94
GO:0045893	positive regulation of DNA-templated transcription	14	1.674	3.94
GO:0008380	RNA splicing	13	1.318	3.66
GO:0000045	autophagosome assembly	7	1.318	1.97
GO:0008286	insulin receptor signaling pathway	6	1.318	1.69
GO:0006357	regulation of transcription by RNA polymerase II	42	1.266	11.83
GO:0006355	regulation of DNA-templated transcription	31	1.266	8.73
GO:0045944	positive regulation of transcription by RNA polymerase II	19	1.266	5.35
GO:0006397	mRNA processing	15	1.266	4.23
GO:0006995	cellular response to nitrogen starvation	4	1.161	1.13
GO:0030154	cell differentiation	24	1.100	6.76
GO:0016567	protein ubiquitination	15	1.100	4.23
GO:0060323	head morphogenesis	3	0.900	0.85
GO:0000423	mitophagy	5	0.818	1.41
GO:0000122	negative regulation of transcription by RNA polymerase II	13	0.674	3.66
GO:0006914	autophagy	7	0.593	1.97
GO:0006417	regulation of translation	7	0.106	1.97
GO:0051028	mRNA transport	5	0.106	1.41
GO:0048745	smooth muscle tissue development	2	0.106	0.56
GO:0043410	positive regulation of MAPK cascade	6	0.070	1.69
GO:0008285	negative regulation of cell population proliferation	4	0.057	1.13
GO:0000381	regulation of alternative mRNA splicing, via spliceosome	4	0.039	1.13
GO:0042554	superoxide anion generation	3	0.039	0.85
GO:1904262	negative regulation of TORC1 signaling	3	0.039	0.85
GO:0014029	neural crest formation	3	0.039	0.85
GO:0048255	mRNA stabilization	3	0.039	0.85
GO:0048566	embryonic digestive tract development	2	0.039	0.56
GO:0016071	mRNA metabolic process	2	0.039	0.56
GO:0070934	CRD-mediated mRNA stabilization	2	0.039	0.56
GO:0010875	positive regulation of cholesterol efflux	2	0.039	0.56
GO:1905564	positive regulation of vascular endothelial cell proliferation	2	0.039	0.56

Group 8

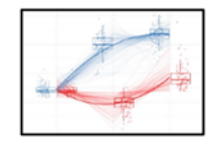

GO ID	GO term	Count	–log10(BH)	%
GO:0006730	one-carbon metabolic process	2	0	3.85
GO:0000423	mitophagy	2	0	3.85
GO:0045595	regulation of cell differentiation	2	0	3.85

Group 9

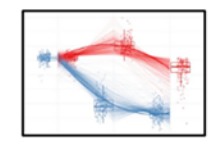

GO ID	GO term	Count	–log10(BH)	%
GO:0051301	cell division	26	15.754	18.18
GO:0007059	chromosome segregation	10	7.283	6.99
GO:0090307	mitotic spindle assembly	5	3.193	3.5
GO:0007080	mitotic metaphase chromosome alignment	4	2.498	2.8
GO:0051225	spindle assembly	5	2.374	3.5
GO:0000070	mitotic sister chromatid segregation	4	2.282	2.8
GO:0009263	deoxyribonucleotide biosynthetic process	3	1.893	2.1
GO:0007018	microtubule-based movement	6	1.735	4.2
GO:0098813	nuclear chromosome segregation	3	1.735	2.1
GO:0000280	nuclear division	3	1.714	2.1
GO:0034501	protein localization to kinetochore	3	1.714	2.1
GO:0006457	protein folding	6	1.642	4.2
GO:0007052	mitotic spindle organization	4	1.333	2.8
GO:0018279	protein N-linked glycosylation via asparagine	3	1.156	2.1
GO:0007094	mitotic spindle assembly checkpoint signaling	3	1.099	2.1
GO:0000086	G2/M transition of mitotic cell cycle	3	1.087	2.1
GO:0007095	mitotic G2 DNA damage checkpoint signaling	3	1.039	2.1
GO:0031536	positive regulation of exit from mitosis	2	0.860	1.4
GO:0051299	centrosome separation	2	0.587	1.4
GO:0000278	mitotic cell cycle	4	0.541	2.8
GO:0051983	regulation of chromosome segregation	2	0.456	1.4
GO:0006260	DNA replication	4	0.429	2.8
GO:0055086	nucleobase-containing small molecule metabolic process	2	0.322	1.4
GO:0043687	post-translational protein modification	2	0.296	1.4
GO:0051315	attachment of mitotic spindle microtubules to kinetochore	2	0.290	1.4
GO:0051256	mitotic spindle midzone assembly	2	0.290	1.4
GO:0008608	attachment of spindle microtubules to kinetochore	2	0.270	1.4
GO:0007088	regulation of mitotic nuclear division	2	0.252	1.4
GO:0030261	chromosome condensation	2	0.236	1.4
GO:0009062	fatty acid catabolic process	2	0.222	1.4
GO:0051231	spindle elongation	2	0.209	1.4
GO:0007064	mitotic sister chromatid cohesion	2	0.197	1.4
GO:0006325	chromatin organization	5	0.149	3.5
GO:0051276	chromosome organization	2	0.149	1.4
GO:0042752	regulation of circadian rhythm	2	0.149	1.4

Group 10

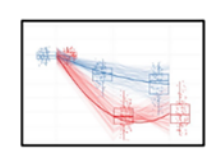

GO ID	GO term	Count	–log10(BH)	%
GO:0050770	regulation of axonogenesis	3	1.396	6.12
GO:0014029	neural crest formation	3	1.144	6.12
GO:0006357	regulation of transcription by RNA polymerase II	10	1.115	20.41
GO:0006355	regulation of DNA-templated transcription	8	1.115	16.33
GO:0009887	animal organ morphogenesis	3	1.115	6.12
GO:0043392	negative regulation of DNA binding	2	1.115	4.08
GO:0050920	regulation of chemotaxis	2	1.115	4.08
GO:0048841	regulation of axon extension involved in axon guidance	2	1.115	4.08
GO:0021637	trigeminal nerve structural organization	2	1.115	4.08
GO:0021785	branchiomotor neuron axon guidance	2	1.115	4.08
GO:0021612	facial nerve structural organization	2	1.115	4.08
GO:0021537	telencephalon development	2	1.115	4.08
GO:0048675	axon extension	2	0.629	4.08
GO:0008045	motor neuron axon guidance	2	0.578	4.08
GO:0030097	hemopoiesis	2	0.479	4.08
GO:0009880	embryonic pattern specification	2	0.471	4.08
GO:0001654	eye development	2	0.403	4.08
GO:0050919	negative chemotaxis	2	0.403	4.08
GO:0007416	synapse assembly	2	0.403	4.08
GO:0045892	negative regulation of DNA-templated transcription	3	0.372	6.12
GO:0032922	circadian regulation of gene expression	2	0.372	4.08
GO:0045944	positive regulation of transcription by RNA polymerase II	4	0.325	8.16
GO:0045893	positive regulation of DNA-templated transcription	3	0.325	6.12
GO:0071526	semaphorin-plexin signaling pathway	2	0.325	4.08

Group 11

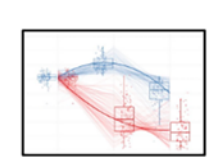

GO ID	GO term	Count	–log10(BH)	%
GO:0098609	cell-cell adhesion	6	2.045	12.24
GO:0030514	negative regulation of BMP signaling pathway	3	0.658	6.12
GO:0000122	negative regulation of transcription by RNA polymerase II	5	0.481	10.2
GO:0045892	negative regulation of DNA-templated transcription	4	0.394	8.16
GO:0006355	regulation of DNA-templated transcription	7	0.051	14.29
GO:0007165	signal transduction	7	0.051	14.29
GO:0030154	cell differentiation	5	0.051	10.2
GO:0007155	cell adhesion	4	0.051	8.16
GO:0007156	homophilic cell-cell adhesion	3	0.051	6.12
GO:0045893	positive regulation of DNA-templated transcription	3	0.051	6.12
GO:0048793	pronephros development	2	0.051	4.08
GO:0045597	positive regulation of cell differentiation	2	0.051	4.08
GO:0007043	cell-cell junction assembly	2	0.051	4.08
GO:0034332	adherens junction organization	2	0.051	4.08
GO:0016339	calcium-dependent cell-cell adhesion	2	0.051	4.08
GO:0044331	cell-cell adhesion mediated by cadherin	2	0.051	4.08

Figure EV3. Related to Figs. 1 and 2.

GO Biological Process (BP) analysis for each gene expression group shown in Fig. 1D. Genes are grouped based on their temporal expression patterns, and GO analysis is performed for the BP category. Tables show selected BP terms for each group, based on statistical ranking and biological relevance, together with the number and percentage of genes associated with each term, and the corresponding $-\log_{10}$(Benjamini-adjusted *P* value). Representative expression patterns for each group are shown above the tables.

 © The Author(s)

A

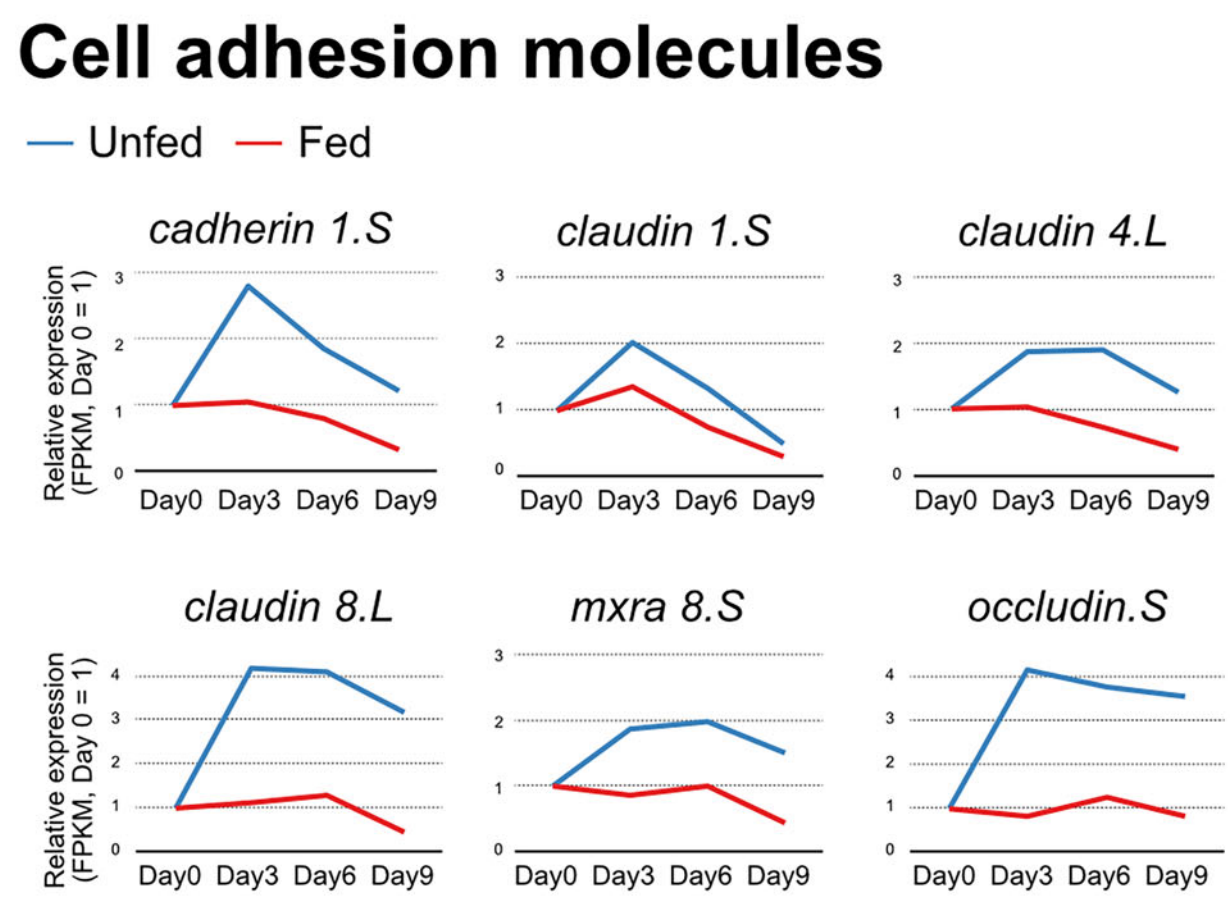

B

Thyroid-related genes

GO:0030878 (Thyroid gland development)
GO:0006590 (Thyroid hormone generation)

**Group 2**
paired box 8 (pax8.L)
carboxypeptidase (cpq.L)
**Group 3**
thyroid peroxidase S homeolog (tpo.S)
**Group 6**
thyroglobulin (tg.L) (LOC108719422)
**Group 4**
endothelin 1 (edn1.S)
**Group 7**
endothelin receptor type A (ednra.S)
v-raf-leukemia viral oncogene 1 (raf1.S)
T-box1 (tbx1.L)
homeobox A3 (hoxa3.S)

Figure EV4. Related to Figs. 1 and 2.

(**A**) Additional graphs are provided for the cell adhesion molecules, showing temporal changes in gene expression as relative expression levels (FPKM, Day 0 = 1). (**B**) Thyroid-related genes and their corresponding groups are summarized.
